# Subterahertz Spin Relaxation Dynamics of Boron-Vacancy Centers in Hexagonal Boron Nitride

**DOI:** 10.1021/acs.nanolett.6c00588

**Published:** 2026-05-29

**Authors:** Abhishek Bharatbhai Solanki, Yueh-Chun Wu, Hamza Ather, Priyo Adhikary, Aravindh Shankar, Ian Gallagher, Xingyu Gao, Owen Matthiessen, Demid Sychev, Alexei Lagutchev, Tongcang Li, Yong P. Chen, Vladimir M. Shalaev, Benjamin Lawrie, Pramey Upadhyaya

**Affiliations:** † Elmore Family School of Electrical and Computer Engineering, 311308 Purdue University, West Lafayette, Indiana 47907, United States; ‡ Birck Nanotechnology Center, 311308 Purdue University, West Lafayette, Indiana 47907, United States; ¶ Materials Science and Technology Division, 6146 Oak Ridge National Laboratory, Oak Ridge, Tennessee 37830, United States; § Department of Physics and Astronomy, 311308 Purdue University, West Lafayette, Indiana 47907, United States; ∥ Purdue Quantum Science and Engineering Institute (PQSEI), 311308 Purdue University, West Lafayette, Indiana 47907, United States; ⊥ Institute of Physics and Astronomy and Villum Centers for Dirac Materials and Hybrid Quantum Materials, Aarhus University, 8000 Aarhus C, Denmark; # WPI-AIMR International Research Center on Materials Sciences, Tohoku University, Sendai 980-8577, Japan

**Keywords:** Quantum sensing, two-dimensional, hexagonal Boron Nitride, subterahertz, high-field magnetometry, boron-vacancies

## Abstract

Quantum sensors based on spin defects have become powerful tools for detecting faint magnetic signals, yet their operation remains confined to low magnetic fields and gigahertz frequencies. Extending such sensors into high-field ( >0.3 T) and subterahertz regimes would enable quantum metrology across a wide range of electromagnetic phenomena and scientific applications, but has proven challenging. Here, we demonstrate that negatively charged boron vacancies $\left(V_{B}^{-}\right)$ in hexagonal boron nitride can function as relaxation-based quantum sensors operating up to 0.2 terahertz and 7 T fields. Their uniform spin-orientation and persistent spin-contrast at high fields enable measurement of intrinsic spin relaxation across unexplored field regimes. We reveal a crossover in relaxation behavior, initially decreasing at low fields before rising at higher fields, consistent with the emergence of single-phonon-induced resonant noise at subterahertz frequencies. These results establish $V_{B}^{-}$ centers as a versatile platform for quantum sensing in the subterahertz, high-field regime.

## Graphical abstract

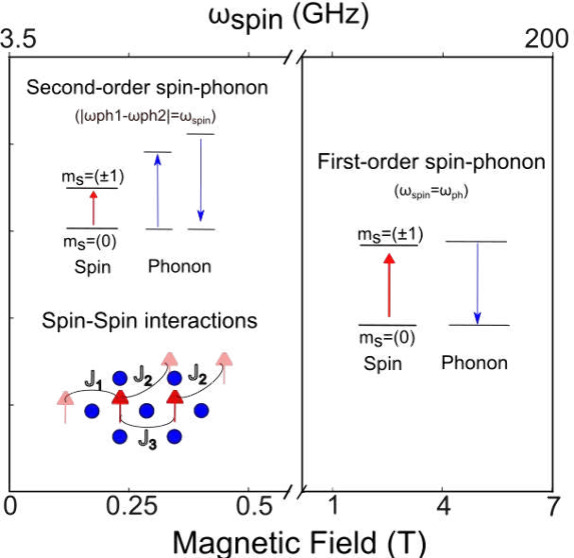

Solid-state spin defects offer a unique combination of optically addressable spin states and long spin coherence times, making them highly attractive for quantum applications.^1^ Quantum sensors based on the relaxation dynamics of spin-defects, most notably the nitrogen-vacancy (NV^–^) center in diamond,^2^ leverage their pronounced sensitivity to environmental magnetic noise,^3–11^ enabling a broad range of scientific applications.^12^ However, the operation of these sensors is generally limited to low magnetic fields ( <0.3 T) —corresponding to low gigahertz (GHz) frequencies. Accessing subterahertz (sub-THz) spin splitting requires precise alignment of Tesla-scale magnetic fields with the defect’s symmetry axis to preserve the optical spin-contrast that forms the basis of spin-state readout techniques.^13–15^ At high magnetic fields, the photodynamics of the NV^–^ center become increasingly complex due to strain-dependent excited-state level anticrossings, which lead to spin mixing.^14^ Furthermore, the fundamental mechanisms governing the longitudinal (*T*_1_) and transverse spin-relaxation (*T*_2_) times at high magnetic fields remain poorly understood, presenting a key challenge for realizing quantum sensing at Tesla-scale fields and subterahertz frequencies.^16^

On another front, recent efforts have increasingly focused on identifying alternative spin-defect platforms,^17–20^ such as ensembles of negatively charged boron-vacancy centers $\left(V_{B}^{-}\right)$ in van der Waals hexagonal boron nitride (hBN).^20^ Unlike their bulk counterparts, $V_{B}^{-}$ spin defects can be engineered in few-layer hBN,^21–24^ enabling unique ultrathin quantum sensors positioned within a few nanometers distance of the target materials. The spin-dipole moment of the $V_{B}^{-}$ center is fixed along the out-of-plane direction, enabling consistent alignment of an external uniaxial magnetic field with all defects in the ensemble. Moreover, their photodynamics remain simple and predictable at high fields,^25^ up to several Teslas, making them particularly attractive candidates for high-field quantum sensing applications. Dense ensembles of coaligned spin defects in few-layer hBN also offer a unique platform to study many-body systems, including the impact of disorder, dimensionality, and dipolar interactions on nanoscale spin-dynamics.^26,27^

In this work, we demonstrate that negatively charged boron vacancies $\left(V_{B}^{-}\right)$ in hBN function as spin–relaxation–based quantum sensors operating in the subterahertz regime. Leveraging this capability, we probe the intrinsic noise mechanisms governing temperature-dependent longitudinal spin relaxation (1/*T*_1_) of $V_{B}^{-}$ centers across magnetic fields from 0 – 7 T ( ∼3.5 GHz–0.2 THz), revealing a rich, nonmonotonic behavior. At low fields (0–0.04 T), where most prior studies have focused, the relaxation rate decreases before saturating. In the higher-field regime (0.17–7 T), it first decreases up to ∼1.8 T, then reverses trend and increases. This nonmonotonicity persists across all temperatures (15–250 K) and strengthens at lower temperatures. A phenomenological model capturing the interplay between phonon-induced and Lorentzian noise (that could be assigned to spin–spin interactions) is introduced to explain these trends. Importantly, we find that the first-order single-phonon (direct) relaxation drives the upturn beyond ∼1.8 T and remains active up to ∼100 K, unlike the low-field case where it is relevant only at millikelvin temperatures.^28^ At higher temperatures, a field-independent two-phonon process starts to dominate, diminishing the nonmonotonicity. Notably, we also observe stretched-exponential relaxation profiles, suggesting complex dynamics dominated by disorder. Together with demonstrated subterahertz relaxometry, these insights establish a microscopic foundation for high-field quantum sensing across a broad range of subterahertz applications.

The ground-state Hamiltonian (*H*_spin_) of $V_{B}^{-}$ defects can be expressed as^29,30^

1 $$H_{spin}=hD\left(S_{z}^{2}-S\left(S+1\right)/3\right)+hE\left(S_{x}^{2}-S_{y}^{2}\right)+g\mu_{B}H_{ext}S_{z}$$

where *D* and *E* denote the axial and transverse zero-field ground state splitting parameters respectively, *h* is Planck’s constant, *g* is the Landé-g factor, μ_B_ is the Bohr Magneton, *S*_*x*,*y*,*z*_ are the spin operators, *S* = 1 for spin-triplet levels, and *H*_ext_ is the external magnetic field aligned with the out-of-plane symmetry axis of the spin defect. At zero field, the ground-state splitting (ZFS) is ∼3.5 GHz and reaches the sub-THz range for several Tesla applied along the quantization axis. When placed in an environment with fluctuating magnetic noise, the spin-defect’s relaxation becomes a probe of the noise resonant with the ground-state splitting. To explore the potential of $V_{B}^{-}$ centers as sub-THz quantum sensors, we use the experimental setup and pulse sequence shown in Figure 1(a) (see methods and Supporting Information Figures S1–S4).

**Figure 1:**
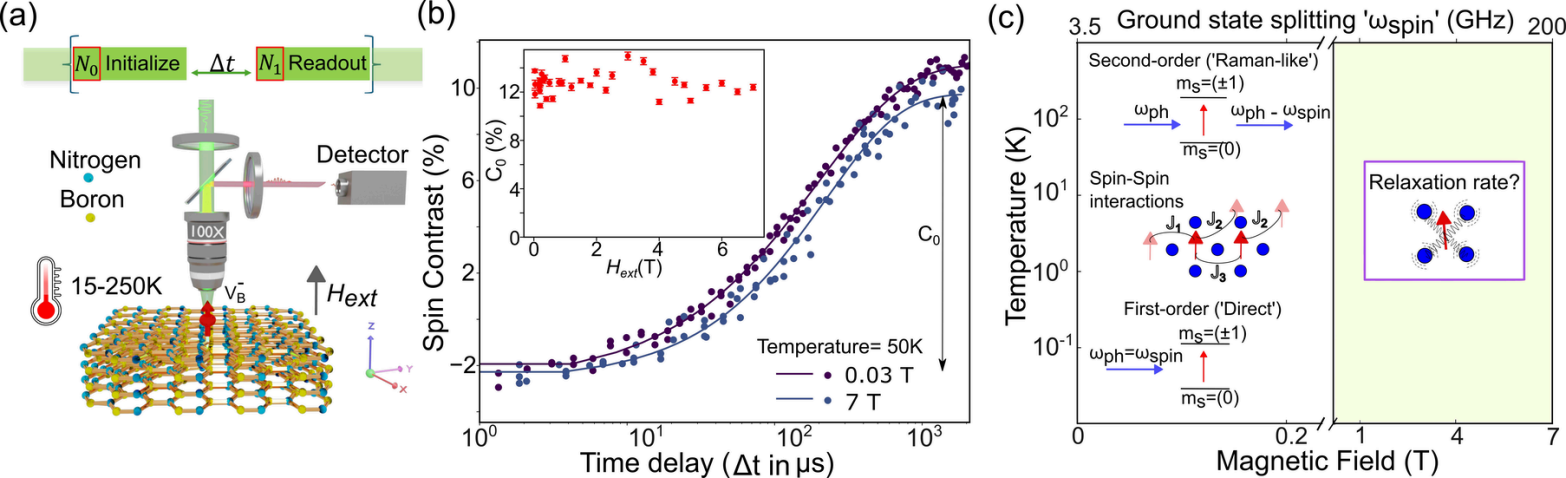
Spin-relaxation mechanisms and measurement scheme (a) The experimental setup comprising a 100*×* microscope objective and a layered hBN sample mounted on the cold-plate of a cryostat with an out-of-plane magnetic field *H*_ext_. The illustrated pulse sequence shows initialization and readout pulses (green) separated by variable delay Δ*t*; PL is measured during the interval highlighted in red. (b) Experimental measurement of the relaxation rate for magnetic fields *H*_ext_ = 0.03, 7 T at a fixed sample temperature *T* = 50 K. The experimental data is overlaid with a stretched exponential fit. Inset: Measured spin-contrast amplitude (*C*_0_) as a function of external magnetic field (*H*_ext_). (c) Schematic diagram illustrating the key spin-relaxation processes in commonly used solid-state spin defects. The upper *x*-axis indicates the ground-state spin splitting (ω_spin_) of a representative spin defect, and the lower *x*-axis shows the corresponding magnetic fields ( ∼200 GHz at 7 T). A dark red arrow represents a central spin-dipole, coupled to surrounding nuclear spins (blue), other identical spin defects, and nearby paramagnetic impurities (light red) with dipolar interaction strength *J*_*i*_. The ground state is also coupled to lattice (ω_ph_) through either resonant first-order (direct) or second-order (Raman-like) spin-phonon processes. The nature of the relaxation dynamics at very high magnetic fields remains largely unexplored.

A 10 μs laser pulse initializes the ground state in the *m*_*s*_ = 0 state, followed by a variable time delay (Δ*t*) and a subsequent readout pulse. The PL signal is measured for a short duration (1.5 μs) at the beginning of both laser pulses. The signal during the readout pulse (*N*_1_) reflects the time-dependent ground-state spin population as the system relaxes toward thermal equilibrium, while the signal measured during initialization (*N*_0_) serves as the reference signal corresponding to the *m*_*s*_ = 0 state. The spin-contrast as a function of time is calculated as $C=\frac{N_{0}-N_{1}}{N_{0}}$ . Representative spin-contrast curves at low (0.03 T) and high fields (7 T) are shown in Figure 1(b). Phenomenologically, the spin-contrast is well described by a stretched exponential $C_{0}\left(1-\exp\left(-{\left(\frac{\Delta t}{T_{1}}\right)}^{\beta }\right)\right)$ ,^31^ where *C*_0_ is the spin-contrast amplitude, Δ*t* is the dark time interval, *T*_1_ is the spin-relaxation time, and β is the stretching factor (see Supporting Information Figures S5–S8). Crucially, *C*_0_ remains large (∼12%) up to the highest field (7 T) studied here (see inset of Figure 1(b)), demonstrating that $V_{B}^{-}$ spin defects can probe their environment’s noise, as encoded in their relaxation rate (1/*T*_1_), in the sub-THz range. Next, we take advantage of this capability to probe the noise environment intrinsic to $V_{B}^{-}$ spin defects across 3.5 GHz–0.2 THz and 15 – 250 K.

Previous works have largely focused on defect spin relaxation at low fields, corresponding to an energy scale significantly smaller than that of typical acoustic phonons.^32–34^ Consequently, the longitudinal spin-relaxation process is primarily governed by second-order spin-phonon coupling mediated by Raman-like scattering involving two high-energy phonons (Figure 1(c)).^32,34,35^ In both $V_{B}^{-}$ centers and NV^–^ centers, this mechanism leads to characteristic temperature dependence of the spin relaxation rate, exhibiting *T* ^2^ and *T* ^5^ scaling behavior, respectively.^32,34^ In contrast, first-order spin-phonon coupling via direct (resonant) scattering of single phonons is relevant at millikelvin temperatures^28,35^ (Figure 1(c)). Dense ensembles of spin defects also exhibit additional relaxation channels arising from dipolar spin–spin interactions between defects and a rapidly fluctuating spin-bath^31,32,36^ (Figure 1(c)). Indeed, relaxation mediated by spin–spin interaction becomes the dominant source of spin relaxation below a critical temperature, where phonon-mediated processes are strongly suppressed.^32^ The goal of the remainder of the article is to address how these mechanisms interplay for $V_{B}^{-}$ centers, particularly in the less studied high-field regime.

In Figure 2(a), we present the experimentally measured relaxation rate $\left(\Gamma =\frac{1}{T_{1}}\right)$ as a function of temperature for several magnetic fields: *H*_ext_ = 0, 0.03, 1.8, 7 T, along with analytical model fits. At temperatures above 125 K, all curves exhibit a *T* ^2^ behavior, characteristic of the two-phonon process.^34^ In contrast, at low temperatures, Γ depends strongly on magnetic field. For low magnetic fields (0.03 T), Γ saturates to a constant value at low temperatures, indicative of a weakly temperature-dependent relaxation mechanism. At an intermediate field (1.8 T), Γ settles at a significantly lower value at low temperatures, suggesting a pronounced magnetic field dependence of the dominant relaxation mechanism. At 7 T, Γ shows a qualitatively different behavior, indicating the emergence of yet another magnetic-field-dependent relaxation mechanism. While prior studies focused on the temperature dependence of Γ at low fields,^34^ our measurements reveal a richer dependence of Γ on magnetic field across the 0 – 7 T range, underscoring the need for a comprehensive study of magnetic-field-dependent relaxation mechanisms.

**Figure 2:**
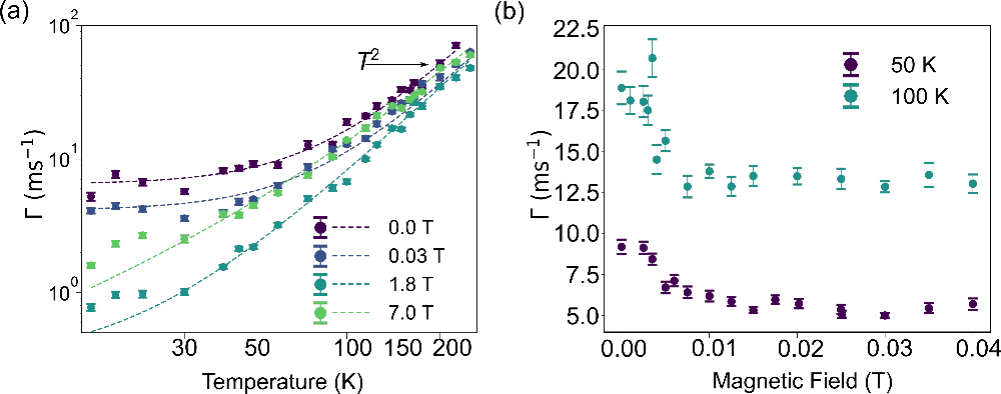
Temperature and magnetic field dependence of the spin relaxation rate of $V_{B}^{-}$ centers in hBN (a) Temperature dependence of the spin relaxation rate (Γ = 1/*T*_1_) measured at fixed external magnetic fields *H*_ext_ = 0, 0.03, 1.8, and 7 T. At high temperatures (*T* > 125 K), all curves follow a *T* ^2^ dependence consistent with a two-phonon Raman-like process. In contrast, the low-temperature behavior exhibits strong field dependence, highlighting the presence of additional relaxation channels. Experimental data are overlaid with analytical model fits. (b) Magnetic field dependence of the relaxation rate for fixed sample temperatures *T* = 50 and 100 K. A sharp suppression of Γ is observed above 0.01 T, attributed to the suppression of resonant spin–spin cross-relaxation, followed by saturation at higher fields.

To this end, we performed magnetic-field-dependent measurement of Γ at several fixed temperatures and variable magnetic fields (*H*_ext_) between 0–7 T. We first focus on the low-field regime, shown in Figure 2(b), which presents data for *H*_ext_ ≤ 0.04 T and temperature *T* = 50, 100 K. In this range, Γ decreases sharply between 0 and 0.01 T and then plateaus to a field-independent value. The rapid suppression of Γ in this field range can be attributed to the reduction of cross relaxation between proximal defects of the same species mediated by dipolar coupling.^32^ At zero or low magnetic fields, the *m*_*s*_ = 0 ↔ ± 1 transitions are close in energy, facilitating nearly resonant cross relaxation between different proximal defects. Applying a modest magnetic field sufficiently separates the energy levels, resulting in a 2-fold reduction in the relaxation rate.^32^ This reduction in Γ could be leveraged to realize an all-optical quantum sensor that is capable of operating at zero or near-zero external magnetic fields.^37^

To gain further insight, we focus on measurements of Γ up to *H*_ext_ = 7 T at several temperatures *T* = 30, 50, 150, 250 K, presented in Figure 3(a, b) along with analytical model fits. The relaxation-rate exhibits a nonmonotonic dependence on magnetic field, with a more pronounced relative change at lower temperatures and a broad minimum centered around 1.8 T. The initial decrease in Γ follows a Lorentzian dependence, reminiscent of spin-relaxation of a central spin caused by a rapidly fluctuating spin-bath.^36^ Beyond 1.8 T, Γ increases up to 7 T, a trend consistent with our previous observation in Figure 2(a). Furthermore, the rate of increase of Γ beyond the lowest point is steeper at elevated temperatures, indicating a role of thermal energy at higher field values. The relaxation rate scales with a characteristic $∼T\cdot {H_{ext}}^{1.6}$ dependence in the high-field regime. Intuitively, this result points to a resonant or ’direct’ process scaled by the number of thermal excitations present in the system, such as spin-relaxation caused by a single phonon. In this scenario, the magnetic field scaling can arise from additional phonon density of states resonant with the ground-state splitting at higher fields.^35^

**Figure 3:**
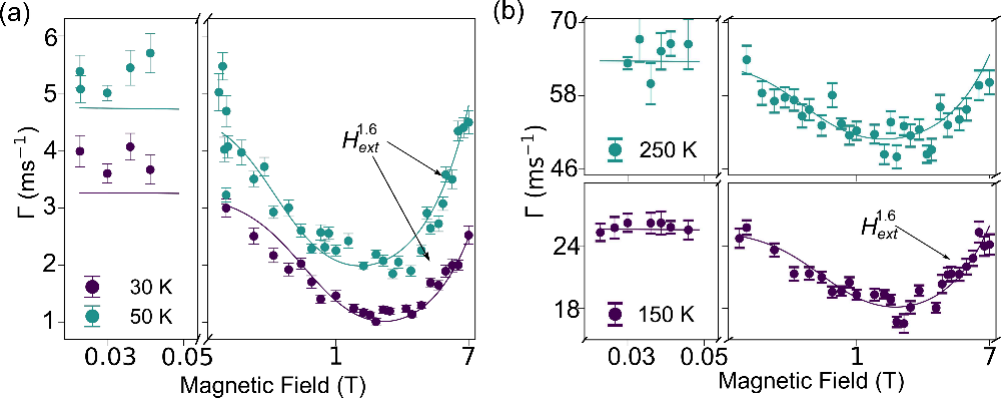
Magnetic-field dependence of the spin relaxation rate of the $V_{B}^{-}$ centers in hBN (a, b) Magnetic field dependence of the relaxation rate $\left(\Gamma =\frac{1}{T_{1}}\right)$ measured at multiple fixed temperatures *T* = 30, 50, 150, and 250 K. Data points represent experimental measurements, and the solid lines show analytical model fits. At low magnetic fields (0.025 < *H*_ext_ < 0.045 T), Γ remains nearly constant, followed by a suppression as the field is increased to 1.8 T. At higher fields ( >1.8 T), Γ increases approximately as $T\cdot H_{ext}^{1.6}$ , consistent with a direct spin-phonon relaxation process. Panels are grouped by temperature range for clarity.

To further elucidate the nature of spin-relaxation processes, we focus on the temperature- and magnetic-field-dependent evolution of the stretching factor (β). In a disordered environment, a stretched exponential decay (β < 1) reflects a broad distribution of relaxation environments, in contrast to a homogeneous case, where β ∼ 1. Although each spin in the ensemble may decay exponentially, the ensemble-averaged signal follows a stretched exponential profile $\left(P\left(t\right)=\exp\left[-{\left(\frac{t}{T_{1}}\right)}^{\beta }\right]\right)$ , with the extent of disorder characterized by β. In Figure 4(a), we present measurements of β versus temperature for *H*_ext_ = 0.03, 7 T and complementary measurements of β versus magnetic field for temperatures *T* = 30, 250 K in Figure 4(b). Across all fields, β consistently decreases as temperature is lowered, with a more pronounced decrease at lower magnetic fields. At low temperatures, β increases monotonically with magnetic field, in contrast to the nonmonotonic field dependence of the relaxation rate.

**Figure 4:**
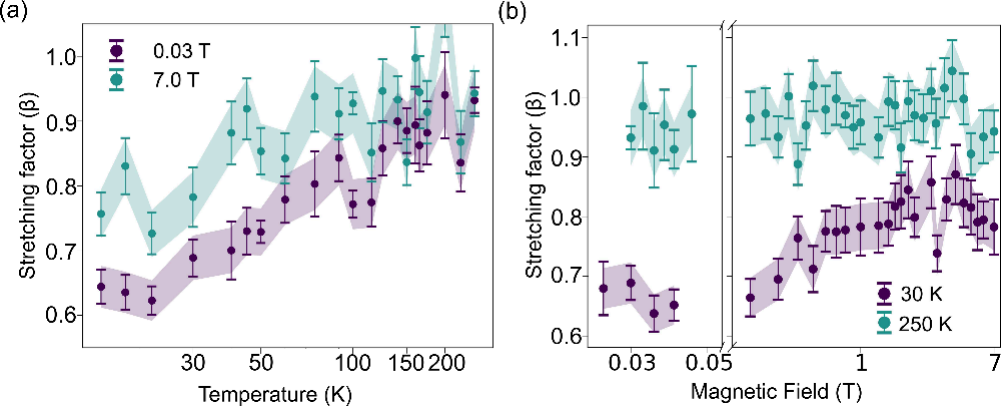
Temperature and magnetic-field dependence of stretching factor (β) (a) Temperature dependence of the stretching factor β for fixed external magnetic fields *H*_ext_ = 0.03, 7 T. (b) Magnetic field dependence of β at fixed sample temperatures *T* = 30 and 250 K, highlighting a pronounced field sensitivity at low temperature and near-constant behavior at high temperature. The evolution of β captures the influence of disorder-induced relaxation processes beyond single-exponential dynamics. Shaded bands represent a fixed ± 5% range around the data points and are used here solely for visualization.

Spin-relaxation in a disordered environment can be influenced by a range of microscopic mechanisms, including fluctuating paramagnetic spins, isotope-disorder, charge dynamics, etc. If spin relaxation is governed solely by local magnetic noise from paramagnetic spins, the stretching factor is β = *d*_spin_/2α, where *d*_spin_ is the dimensionality of the dipolar spin–spin interactions and α characterizes the long-range magnetic dipolar interaction power law, yielding β = 0.5 for a three-dimensional ensemble.^27,31,38^ Additionally, isotope variation and local strain can create inhomogeneities in the spin-phonon coupling, further contributing to nonuniform spin-relaxation dynamics across the measurement area. Moreover, our all-optical measurement scheme may introduce unintended charge dynamics via laser excitation,^39,40^ leading to a multiexponential relaxation mimicking stretched exponential decay. To assess the role of charge dynamics, we performed differential measurements at low magnetic fields using a π-pulse inversion scheme to cancel out nonspin-related contributions. Notably, these measurements also exhibit stretched exponential profiles, suggesting that charge dynamics are unlikely to be the primary source of the observed stretched exponential behavior (see Supporting Information Figure S9). Nonetheless, we note that stretched exponential dynamics cannot be definitively attributed to a single origin without further measurements.

Taken together, the dependence of Γ and β on magnetic field points to a crossover in relaxation dynamics as the field approaches ∼1.8 T. Motivated by these observations, we propose a potential phenomenological model that captures the experimental trends, beginning with spin-phonon interactions.

In our experiments, the ground-state splitting (*ℏω*_0_ ∼ *D* ± *gμ*_B_ *H*_ext_) spans 0 – 0.2 THz across the measurement range (0–7 T), which corresponds to the linear branch of acoustic phonons in the host material hBN. Within this energy range, first-order spin-phonon relaxation is mediated by resonant absorption and emission of phonons (ω_ph_ = ω_0_).^35^ In contrast, second-order spin-phonon relaxation involves nonresonant Raman-like scattering of two higher-energy phonons (ω_*ph*1_, ω_*ph*2_), whose energy difference satisfies ω_*ph*1_ – ω_*ph*2_ = ω_0_, resulting in a spin-flip transition.^35^ Within a Debye-like model, the combined relaxation rate due to spin-phonon coupling can be expressed as

2 $$\Gamma^{\mathrm{spin}‐\mathrm{ph}}=A_{1}\cdot T\cdot \omega_{0}^{n_{1}}+A_{2}\cdot T^{n_{2}}$$

where *A*_1_, *A*_2_ are coupling constants, *T* is the sample temperature, and *n*_1_, *n*_2_ are scaling parameters.^32,34,35,41,42^

The first term is motivated by the first-order spin-phonon relaxation process. The linear *T* dependence accounts for the thermal distribution of phonon modes (in the limit *ℏω*_0_ < *k*_*B*_ *T*) resonant with ω_0_. For low-energy acoustic phonons, the effective density of states of the phonon-bath and the strength of the linear spin-phonon coupling are modeled by a scaling law $\left(\omega_{0}^{n_{1}}\right)$ .^35^ This analytical expression reproduces the observed increase in Γ following its minimum near 1.8 T (for *n*_1_ > 0), highlighting that first-order spin-phonon coupling becomes critical in our case, where large external magnetic fields push ω_0_ into a regime where resonant processes are significantly enhanced. We note that deviations from a simple Debye-like model can yield more complex scaling behavior across the frequency range, requiring first-principle investigation.^43^

The second term accounts for the second-order spin-phonon relaxation process. Previous studies combining first-principle calculations and experiments at low magnetic fields have identified phonon modes near 18 meV as the dominant contributors to the second-order nonresonant spin-relaxation process (ω_*ph*1_ – ω_*ph*2_ = ω_0_).^34^ These phonon modes are substantially higher in energy compared to the typical ground state splitting of $V_{B}^{-}$ centers^34,35^ (see Supporting Information for the phonon spectrum). In contrast to the first-order term, the second-order spin-phonon coupling is modeled to be independent of the magnetic field due to its nonresonant nature and depends solely on temperature $\left(T^{n_{2}}\right)$ .^35,41,42^

Next, we model the initial decrease in Γ as the field is increased to 1.8 T. As mentioned before, this feature is empirically described by a Lorentzian noise model. In previous studies, this mathematical framework has been used to describe the coupling of a central spin with a spin bath consisting of randomly fluctuating spins with a characteristic correlation time (τ_c_).^36^ The density of $V_{B}^{-}$ centers in our sample is estimated to be ∼150ppm (see Supporting Information), corresponding to an average interdefect spacing of ∼4 –5 nm, where dipolar interactions between proximal defects are expected to be non-negligible.^44^ In this regime, faster-relaxing spins including $V_{B}^{-}$ centers (e.g., due to local charge fluctuations) or implantation-induced paramagnetic impurities (e.g., *S* = 1/2 centers),^45,46^ can act as effective magnetic noise sources, driving relaxation of nearby spins via dipolar fields.^31^ The effectiveness of this mechanism depends on the spectral overlap between the ground state splitting (ω_0_) and the magnetic noise power spectrum of the spin bath (*S*(ω)). The resulting relaxation rate takes the form

3 $$\Gamma^{\mathrm{Lorentzian}}=\gamma^{2}S\left(\omega_{0}\right)=\frac{\eta \cdot \tau_{c}}{1+{\left(\omega_{0}\tau_{c}\right)}^{2}}$$

where γ is the gyromagnetic ratio and η­(*T*) is a phenomenological constant that captures the density and dipolar coupling strength of the fluctuating spins. The parameters η­(*T*) and τ_c_(*T*) serve as fit parameters, providing insight into the Lorentzian bath properties.

Based on the physical picture outlined above, we fit the magnetic field-dependent and temperature-dependent relaxation rate (Γ) with a combined analytical model Γ­(*H*_ext_, *T*) = Γ^Lorentzian^ + Γ^spin‑ph^ to estimate the relative contributions of the different relaxation mechanisms. A representative fit of the magnetic field dependent data at *T* = 100 K, along with contributions from all mechanisms, is presented in Figure 5(a). At low fields, the relaxation is dominated by Γ^Lorentzian^. Beyond 1.8 T, the field-dependent first-order spin-phonon relaxation term grows in magnitude, leading to an observable rise in Γ. In contrast, the second-order spin-phonon term manifests as a constant background contribution at a fixed temperature. At elevated temperatures, the contribution from the second-order term is higher, thereby reducing the relative variation of Γ with *H*_ext_. Notably, this fitting procedure yields an effective exponent *n*_1_ ∼ 1.6, implying that at higher magnetic fields, where ω_0_ ∝ *H*_ext_, the effective first-order spin-phonon relaxation rate scales as $∼H_{ext}^{1.6}$ .

**Figure 5:**
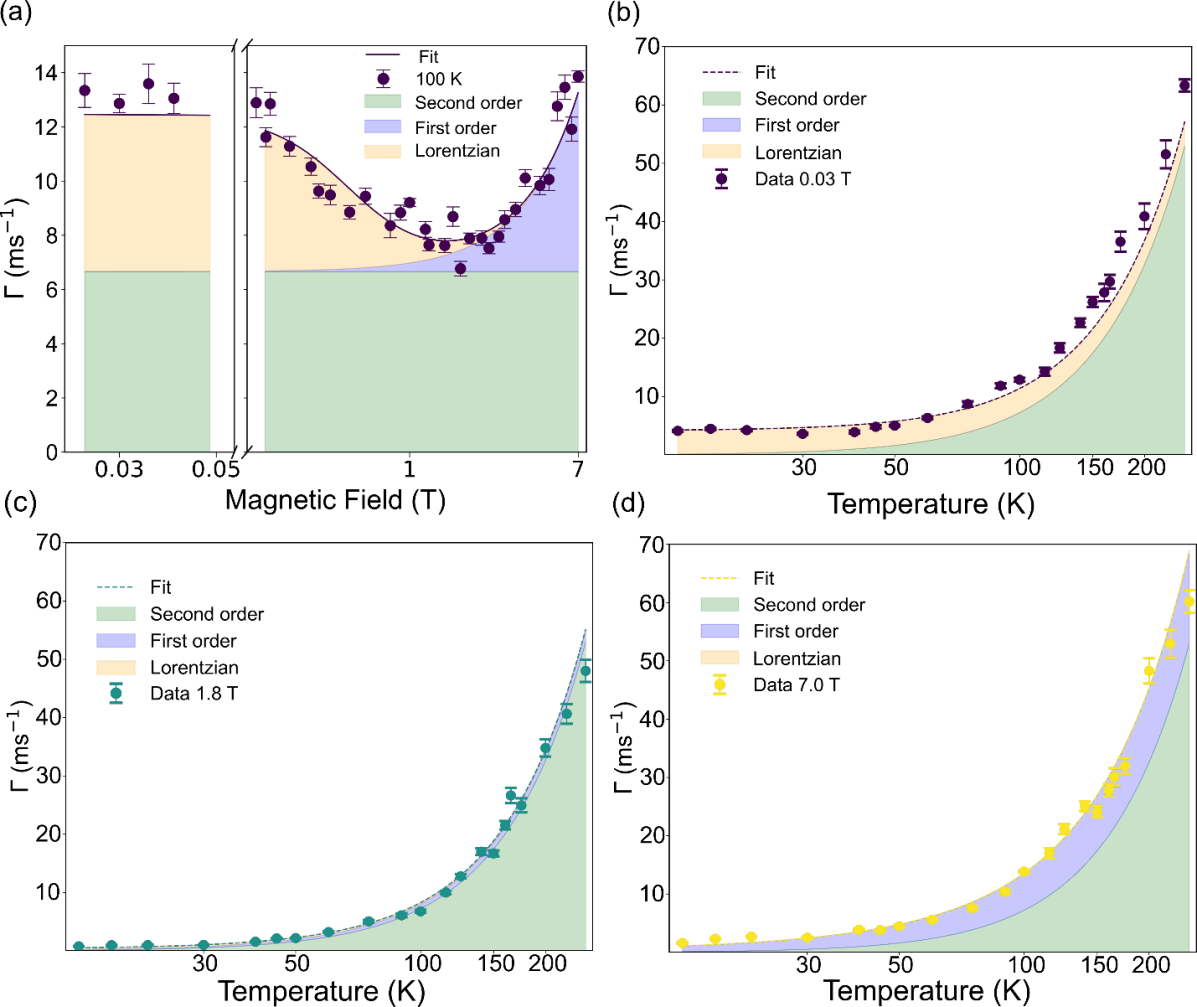
Temperature and magnetic field dependence of the spin relaxation rate (Γ = 1/*T*_1_) of $V_{B}^{-}$ centers in hBN, along with contributions from individual relaxation mechanisms. (a) Magnetic field dependence of Γ at *T* = 100 K, showing nonmonotonic behavior with a minimum near 1.8 T. (b–d) Temperature-dependent relaxation rates at selected magnetic fields: (b) 0.03 T, (c) 1.8 T, and (d) 7 T. Experimental data are overlaid with model fits (dashed lines), decomposed into contributions from second-order spin-phonon (green), first-order spin-phonon (purple), and Lorentzian (orange) processes. Stacked shaded regions correspond to the same individual mechanisms as in (b–d). The consistent modeling across both temperature and field dependencies highlights the interplay of multiple relaxation channels in determining the total spin relaxation rate.

To further validate our analytical model, we present temperature-dependent fits, along with the individual contributions from all mechanisms at fixed magnetic fields *H*_*ext*_ = 0.03, 1.8, and 7 T in Figure 5(b–d). These fits reveal similar trends: at low magnetic fields, the relaxation is governed by the Lorentzian noise source, while at higher fields and elevated temperatures, spin-phonon processes become dominant (see additional data in Supporting Information Figure S10). This analysis yields an exponent *n*_2_ ∼ 2 for all magnetic fields, in agreement with previously observed *T* ^2^ scaling for experiments at low fields.^34^ The analytical fits obtained from our model show excellent agreement with the experimental trends in the entire parameter space. The extracted parameters are consistent between fits to magnetic field and temperature-dependent data, lending further support to the reliability of our analytical framework.

While the present model highlights the dominant role of single-phonon processes at high fields, a complete microscopic understanding of spin–phonon interactions requires going beyond the simple Debye-like framework. For instance, the observed nonmonotonicity may reflect scaling behaviors beyond those captured in eq 2, potentially arising from more complex phonon spectral functions,^43^ as well as contributions from flexural phonon modes, interlayer breathing modes, and their coupling to the substrate. In addition, relaxation channels largely independent of temperature and magnetic field, such as dipolar interactions between resonant $V_{B}^{-}$ defects or charge dynamics, may also contribute to the measured rates. These factors complicate the quantitative extraction of coupling constants and bath properties from the present data. A comprehensive microscopic understanding will require first-principles modeling and targeted experiments, such as thickness and doping dependence, which lie beyond the scope of the present study.

In conclusion, leveraging the robust spin-contrast at 7 T field, we demonstrate that the relaxation of $V_{B}^{-}$ spin defects can be measured over frequencies ranging from GHz to sub-THz regimes. Exploiting this functionality, we probe relaxation mechanisms intrinsic to $V_{B}^{-}$ defects across a broad frequency and temperature range. The longitudinal relaxation rate of $V_{B}^{-}$ centers exhibits a pronounced dependence on external magnetic field, highlighting the complex interplay between multiple relaxation mechanisms. While first-order spin-phonon interactions have traditionally been considered important only at extremely low temperatures, our results reveal the crucial role they play at elevated temperatures up to 100 K at higher magnetic fields. The measured relaxation profiles also exhibit a stretched exponential decay, indicative of disorder in the system. We also find that the longest spin relaxation time is achieved near ∼1.8 T and ∼15 –20 K, with a maximum *T*_1_ ∼ 1.3 ms, comparable to reported *T*_1_ times in NV centers in nanodiamonds.^47^

Our experiments open exciting possibilities for designing relaxation-based quantum sensors capable of operating at fields as high as 7 T, corresponding to an energy scale of ∼0.2 THz. Accessing this sub-THz regime is considered challenging due to a lack of reliable sources and detectors.^48^ In this context, the all-optical measurement scheme employed in our experiments offers a promising route to probe sub-THz phenomena, such as magnetic modes in antiferromagnets.^49,50^ High-field magnetometry enabled by such sensors can be used to investigate exotic condensed-matter phases at several Teslas.^51^ Furthermore, these experiments can be extended to even higher magnetic fields for $V_{B}^{-}$ defects and other spin-defect species,^52–54^ and towards coherent control using emerging approaches such as RF delivery over optical fibers.^55^ More broadly, these findings lay the foundation for exploring many-body dynamics in dense ensembles of $V_{B}^{-}$ defects. They also provide key insights into the fundamental relaxation and decoherence mechanisms of $V_{B}^{-}$ defects at high magnetic fields, a crucial step in tailoring these defects for quantum applications.

## Supplementary Material



## Data Availability

The data that support the findings of this study are available from the corresponding author upon reasonable request.

## Notes

Methods; flake characterization; analytical fits of the experimental relaxation data; microwave-assisted differential measurements; data analysis and fitting; first-principles calculation of the phonon spectrum; additional temperature-dependent data (PDF)

## References

[1] Wolfowicz G., Heremans F. J., Anderson C. P., Kanai S., Seo H., Gali A., Galli G., Awschalom D. D. (2021). Quantum
guidelines for solid-state spin defects. Nature
Reviews Materials.

[2] Doherty M., Manson N., Delaney P., Jelezko F., Wrachtrup J., Hollenberg L. (2013). The nitrogen-vacancy colour centre
in diamond. Phys. Rep..

[3] Thiel L., Wang Z., Tschudin A., Rohner D., Gutiérrez-Lezama I., Ubrig N., Gibertini M., Giannini E., Morpurgo F., Maletinsky P. (2019). Probing magnetism
in 2D materials at the nanoscale
with single-spin microscopy. Science.

[4] Machado F., Demler E., Yao N., Chatterjee S. (2023). Quantum Noise
Spectroscopy of Dynamical Critical Phenomena. Phys. Rev. Lett..

[5] Curtis J. B., Maksimovic N., Poniatowski N. R., Yacoby A., Halperin B., Narang P., Demler E. (2024). Probing the Berezinskii-Kosterlitz-Thouless
vortex unbinding transition in two-dimensional superconductors using
local noise magnetometry. Phys. Rev. B.

[6] Ziffer, M. E. Quantum Noise Spectroscopy of Criticality in an Atomically Thin Magnet. arXiv 2024, arXiv:2407.05614v2, submitted 2024-08-15 (accessed 2026-03-25).

[7] Casola F., van der Sar T., Yacoby A. (2018). Probing condensed matter physics
with magnetometry based on nitrogen-vacancy centres in diamond. Nature Reviews Materials.

[8] Flebus B., Ochoa H., Upadhyaya P., Tserkovnyak Y. (2018). Proposal for
dynamic imaging of antiferromagnetic domain wall via quantum-impurity
relaxometry. Phys. Rev. B.

[9] Chatterjee S., Rodriguez-Nieva J. F., Demler E. (2019). Diagnosing phases of
magnetic insulators
via noise magnetometry with spin qubits. Phys.
Rev. B.

[10] Solanki A., Bogdanov S., Rahman M., Rustagi A., Dilley N. R., Shen T., Tong W., Debashis P., Chen Z., Appenzeller J., Chen Y., Shalaev V., Upadhyaya P. (2022). Electric field
control of interaction between magnons and quantum spin defects. Physical Review Research.

[11] Wu Y.-C., Halasz G. B., Damron J. T., Gai Z., Zhao H., Sun Y., Dahmen K. A., Sohn C., Carlson E. W., Hua C., Lin S., Song J., Lee H. N., Lawrie B. J. (2025). Nanoscale magnetic
ordering dynamics in a high Curie temperature ferromagnet. Nano Lett..

[12] Degen C., Reinhard F., Cappellaro P. (2017). Quantum Sensing. Rev. Mod. Phys..

[13] Hopper D., Shulevitz H., Bassett L. (2018). Spin readout techniques of the nitrogen-vacancy
center in diamond. Micromachines.

[14] Happacher J., Broadway D. A., Bocquel J., Reiser P., Jimenéz A., Tschudin M. A., Thiel L., Rohner D., Puigibert M. l. G., Shields B., Maze J. R., Jacques V., Maletinsky P. (2022). Low-Temperature
Photophysics of Single Nitrogen-Vacancy Centers in Diamond. Phys. Rev. Lett..

[15] Sahin O., de Leon Sanchez E., Conti S., Akkiraju A., Reshetikhin P., Druga E., Aggarwal A., Gilbert B., Bhave S., Ajoy A. (2022). High field magnetometry with hyperpolarized nuclear spins. Nat. Commun..

[16] Kollarics S., Markus B. G., Kucsera R., Thiering G., Gali A., Nemeth G., Kamaras K., Forro L., Simon F. (2024). Terahertz
emission from diamond nitrogen-vacancy centers. Science Advances.

[17] Raha M., Chen S., Phenicie C., Ourari S., Dibos A., Thompson J. (2020). Optical quantum nondemolition measurement of a single
rare earth ion qubit. Nat. Commun..

[18] Bradac C., Gao W., Forneris J., Trusheim M. E., Aharonovich I. (2019). Quantum nanophotonics
with group IV defects in diamond. Nat. Commun..

[19] Koehl W., Buckley B., Heremans F., Calusine G., Awschalom D. (2011). Room temperature
coherent control of defect spin qubits in silicon carbide. Nature.

[20] Vaidya, S. ; Gao, X. ; Dikshit, S. ; Aharonovich, I. ; Li, T. Quantum sensing and imaging with spin defects in hexagonal boron nitride. Advances in Physics: X 2023, 8. 10.1080/23746149.2023.2206049.

[21] Gao X., Jiang B., Llacsahuanga Allcca A.
E., Shen K., Sadi M. A., Solanki A. B., Ju P., Xu Z., Upadhyaya P., Chen Y. P., Bhave S. A., Li T. (2021). High-Contrast
Plasmonic-Enhanced Shallow Spin Defects in Hexagonal Boron Nitride
for Quantum Sensing. Nano Lett..

[22] Xu X. (2023). Greatly Enhanced Emission
from Spin Defects in Hexagonal Boron Nitride
Enabled by a Low-Loss Plasmonic Nanocavity. Nano Lett..

[23] Durand A., Clua-Provost T., Fabre F., Kumar P., Li J., Edgar J. H., Udvarhelyi P., Gali A., Marie X., Robert C., Gérard J. M., Gil B., Cassabois G., Jacques V. (2023). Optically Active Spin Defects in Few-Layer Thick Hexagonal
Boron Nitride. Phys. Rev. Lett..

[24] Zhou J., Lu H., Chen D., Huang M., Yan G. Q., Al-matouq F., Chang J., Djugba D., Jiang Z., Wang H., Du C. R. (2024). Sensing spin wave excitations by spin defects in few-layer-thick
hexagonal boron nitride. Science Advances.

[25] Clua-Provost T. (2024). Spin-dependent photodynamics
of boron-vacancy centers in hexagonal
boron nitride. Phys. Rev. B.

[26] Zu C., Machado F., Ye B., Choi S., Kobrin B., Mittiga T., Hsieh S., Bhattacharyya P., Markham M., Twitchen D., Jarmola A., Budker D., Laumann C. R., Moore J. E., Yao N. Y. (2021). Emergent hydrodynamics
in a strongly interacting dipolar spin ensemble. Nature.

[27] Davis E. J. (2023). Probing many-body dynamics in a two-dimensional dipolar spin ensemble. Nat. Phys..

[28] Astner T., Gugler J., Angerer A., Wald S., Putz S., Mauser N. J., Trupke M., Sumiya H., Onoda S., Isoya J., Schmiedmayer J., Mohn P., Majer J. (2018). Solid-state
electron spin lifetime limited by phononic vacuum modes. Nat. Mater..

[29] Gottscholl A., Kianinia M., Soltamov V., Orlinskii S., Mamin G., Bradac C., Kasper C., Krambrock K., Sperlich A., Toth M., Aharonovich I., Dyakonov V. (2020). Initialization and read-out of intrinsic spin defects
in a van der Waals crystal at room temperature. Nat. Mater..

[30] Gottscholl A., Diez M., Soltamov V., Kasper C., Sperlich A., Kianinia M., Bradac C., Aharonovich I., Dyakonov V. (2021). Room temperature coherent control of spin defects in
hexagonal boron nitride. Science Advances.

[31] Choi J., Choi S., Kucsko G., Maurer P., Shields B., Sumiya H., Onoda S., Isoya J., Demler E., Jelezko F., Yao N., Lukin M. (2017). Depolarization Dynamics
in a Strongly Interacting Solid-State Spin Ensemble. Phys. Rev. Lett..

[32] Jarmola A., Acosta V. M., Jensen K., Chemerisov S., Budker D. (2012). Temperature- and Magnetic-Field-Dependent Longitudinal
Spin Relaxation in Nitrogen-Vacancy Ensembles in Diamond. Physical Review Research.

[33] Liu W. (2021). Temperature-Dependent
Energy-Level Shifts of Spin Defects in Hexagonal
Boron Nitride. ACS Photonics.

[34] Liu Z., Gong R., Huang B., Jin Y., Du X., He G., Janzen E., Yang L., Henriksen E. A., Edgar J. H., Galli G., Zu C. (2025). Temperature-dependent
spin-phonon coupling of boron-vacancy centers in hexagonal boron nitride. Phys. Rev. B.

[35] Norambuena A., Muñoz E., Dinani H. T., Jarmola A., Maletinsky P., Budker D., Maze J. R. (2018). Spin-lattice relaxation of individual
solid-state spins. Phys. Rev. B.

[36] de
Guillebon T., Vindolet B., Roch J.-F., Jacques V., Rondin L. (2020). Temperature dependence of the longitudinal spin relaxation
time *T*
_1_ of single nitrogen-vacancy centers
in nanodiamonds. Phys. Rev. B.

[37] Pellet-Mary C., Perdriat M., Huillery P., Hétet G. (2023). Relaxation
Processes in Dipole-Coupled Nitrogen-Vacancy Centers in Zero Field:
Application in Magnetometry. Phys. Rev. Appl..

[38] Kucsko G., Choi S., Choi J., Maurer P. C., Zhou H., Landig R., Sumiya H., Onoda S., Isoya J., Jelezko F., Demler E., Yao N. Y., Lukin M. D. (2018). Critical
Thermalization of a Disordered Dipolar Spin System in Diamond. Phys. Rev. Lett..

[39] Giri R., Gorrini F., Dorigoni C., Avalos C. E., Cazzanelli M., Tambalo S., Bifone A. (2018). Coupled charge and spin dynamics
in high-density ensembles of nitrogen-vacancy centers in diamond. Phys. Rev. B.

[40] Cardoso
Barbosa I., Gutsche J., Widera A. (2023). Impact of charge conversion
on NV-center relaxometry. Phys. Rev. B.

[41] Cambria M. C., Gardill A., Li Y., Norambuena A., Maze J. R., Kolkowitz S. (2021). State-dependent
phonon-limited spin
relaxation of nitrogen-vacancy centers. Physical
Review Research.

[42] Cambria M. C., Norambuena A., Dinani H. T., Thiering G., Gardill A., Kemeny I., Li Y., Lordi V., Gali Á., Maze J. R., Kolkowitz S. (2023). Temperature-Dependent Spin-Lattice
Relaxation of the Nitrogen-Vacancy Spin Triplet in Diamond. Phys. Rev. Lett..

[43] Lunghi A., Sanvito S. (2019). How do phonons relax molecular spins?. Science Advances.

[44] Gong R., He G., Gao X., Ju P., Liu Z., Ye B., Henriksen E. A., Li T., Zu C. (2023). Coherent dynamics of
strongly interacting electronic spin defects in hexagonal boron nitride. Nat. Commun..

[45] Haykal A., Tanos R., Minotto N., Durand A., Fabre F., Li J., Edgar J. H., Ivády V., Gali A., Michel T., Dréau A., Gil B., Cassabois G., Jacques V. (2022). Decoherence of V_B̂_-spin defects in
monoisotopic hexagonal boron nitride. Nat. Commun..

[46] Vlassiouk I. V., Wu Y.-C., Puretzky A., Liang L., Lasseter J., Dryzhakov B., Gallagher I., Ghosh S., Lavrik N., Dyck O., Lupini A. R., Checa M., Collins L., Meyer H. M., Zhao H., Likhi F., Xiao K., Ivanov I., Glasgow D., Tselev A., Lawrie B., Smirnov S., Randolph S. J. (2026). Defect Engineering in Large-Scale
CVD-Grown Hexagonal Boron Nitride: Formation, Spectroscopy, and Spin
Relaxation Dynamics. Small.

[47] Benedičič I., Tanuma Y., Gosar i. c. v., Anézo B., Mrózek M., Wojciechowski A., Arčon D. (2025). Spin-lattice
relaxation of NV centers in nanodiamonds adsorbed on conducting and
nonconducting surfaces. Phys. Rev. B.

[48] Herter A., Shams-Ansari A., Settembrini F., Warner H. K., Faist J., Loncar M., Benea-Chelmus I.-C. (2023). Terahertz waveform synthesis in integrated
thin-film lithium niobate platform. Nat. Commun..

[49] Baltz V., Manchon A., Tsoi M., Moriyama T., Ono T., Tserkovnyak Y. (2018). Antiferromagnetic
spintronics. Rev. Mod. Phys..

[50] Rimmler B., Pal B., Parkin S. (2025). Non-collinear antiferromagnetic
spintronics. Nature Reviews Materials.

[51] Banerjee A., Yan J., Knolle J., Bridges C. A., Stone M. B., Lumsden M. D., Mandrus D. G., Tennant D. A., Moessner R., Nagler S. E. (2017). Neutron
scattering in the proximate quantum spin liquid *α*-RuCl_3_. Science.

[52] Stern H. L., Gu Q., Jarman J., Eizagirre Barker S., Mendelson N., Chugh D., Schott S., Tan H. H., Sirringhaus H., Aharonovich I., Atature M. (2022). Room-temperature optically detected
magnetic resonance of single defects in hexagonal boron nitride. Nat. Commun..

[53] Stern H. L., Gilardoni C. M., Gu Q., Barker S. E., Powell O. F. J., Deng X., Fraser S. A., Follet L., Li C., Ramsay A. J., Tan H. H., Aharonovich I., Atatüre M. (2024). A quantum coherent spin in hexagonal
boron nitride
at ambient conditions. Nat. Mater..

[54] Gao X., Vaidya S., Li K., Ge Z., Dikshit S., Zhang S., Ju P., Shen K., Jin Y., Ping Y., Li T. (2025). Single nuclear spin detection and
control in a van der Waals material. Nature.

[55] Rahman, M. R. ; Schnier, K. ; Goldsmith, R. ; Lawrie, B. ; Lukens, J. ; Kim, S. M. ; Kung, P. Photonic Links for Spin-Based Quantum Sensors. arXiv, 2026, 2601.22011v1, submitted on 2026-01-29 (accessed on 2026-03-25).

