# Supplementary material for: Subterahertz Spin Relaxation Dynamics of Boron-Vacancy Centers in Hexagonal Boron Nitride
Source: Nano Lett. 2026 May 29;26(22):7296–303. doi: 10.1021/acs.nanolett.6c00588 (PMC13267176; doi:10.1021/acs.nanolett.6c00588)
Supplement: Supplementary file 1 [file nl6c00588_si_002.pdf]

Sub-terahertz Spin Relaxation Dynamics of Boron-Vacancy Centers in Hexagonal Boron Nitride (Supplementary Information)

Abhishek Bharatbhai Solanki,[†,‡] Yueh-Chun Wu,[¶] Hamza Ather,[‡,§] Priyo Adhikary,[†] Aravindh Shankar,[†] Ian Gallagher,[¶] Xingyu Gao,[§] Owen Matthiessen,[†,‡] Demid Sychev,[†,‡] Alexei Lagutchev,[‡] Tongcang Li,[†,‡,§,‖] Yong P. Chen,[†,‡,§,‖,⊥,#] Vladimir M. Shalaev,[*,†,‡,§,‖] Benjamin Lawrie,[*,¶] and Pramey Upadhyaya[*,†,‖]

†*Elmore Family School of Electrical and Computer Engineering, Purdue University, West Lafayette, IN 47906, USA*

‡*Birck Nanotechnology Center, Purdue University, West Lafayette, IN 47906, USA*

¶*Materials Science and Technology Division, Oak Ridge National Laboratory, Oak Ridge, TN 37830, USA*

§*Department of Physics and Astronomy, Purdue University, West Lafayette, IN 47906, USA*

‖*Purdue Quantum Science and Engineering Institute (PQSEI), Purdue University, West Lafayette, IN 47906, USA*

⊥*Institute of Physics and Astronomy and Villum Centers for Dirac Materials and Hybrid Quantum Materials, Aarhus University, 8000 Aarhus-C, Denmark*

#*WPI-AIMR International Research Center on Materials Sciences, Tohoku University, Sendai 980-8577, Japan*

E-mail: shalaev@purdue.edu; lawriebj@ornl.gov; prameyup@purdue.edu

Methods

A single crystal of hexagonal Boron Nitride (hBN) with naturally abundant nuclear isotope distribution was mechanically exfoliated onto a standard SiO_2/Si substrate. The entire substrate, containing flakes of varying thicknesses, was implanted using helium ions, at a dosage of 1 ion/nm^2 at 2.8 keV. Suitable hBN flakes were identified using an optical microscope and transferred to a fresh Si substrate to avoid background fluorescence from the damaged Si substrate, using standard pick-up and transfer procedure. A suitable hBN flake ($\sim$ 42 nm in thickness) was identified post-transfer and characterized with room-temperature optical measurements (see Supplementary Information Figures S1-S4). All primary relaxation measurements reported in the main text (Figure 2,3,4,5) were performed on hBN flakes on a standard SiO_2/Si substrate, without the presence of any metallic structures in the vicinity of the optical excitation region. This configuration ensures that the measured spin relaxation dynamics are not influenced by Johnson noise or other artifacts associated with nearby conductive elements.

Cryogenic experiments at Purdue University and Oak Ridge National Laboratory were performed using an Opticool cryostat from Quantum Design. The cryostat is equipped with a 7 T superconducting magnet, with the magnetic field aligned along the out-of-plane direction relative to the sample. Optical measurements were performed with a 532 nm laser from Hubner Photonics. The excitation beam was incident normal to the sample surface, with both excitation and collection performed through a $100x$ in-vacuum objective. The excitation beam was scanned across the sample using a 4f system with a galvoscanner. The resultant photoluminescence (PL) signal was detected using a Si-based single-photon-avalanche-detector (SPAD) from Thorlabs. The PL signal was filtered with a 700 nm long-pass filter to selectively detect emission from V_B^- defects. The laser beam was modulated by an acousto-optic modulator (AOM) to generate $\sim 10\mu s$ laser pulses for spin initialization and readout. The SPAD output was recorded via a gated counter in a National Instruments (NI) data acquisition card (DAQ), with a counter window duration of $1.5\mu s$. A pulse streamer

8/2 from Swabian Instruments was used to synchronize the experiment.

Microwave measurements were performed in separate control experiments (Supplementary Information Figure S9) using a gold waveguide (300 nm thick) patterned onto a sapphire substrate. These measurements were designed to implement a differential scheme to isolate and eliminate contributions from charge dynamics. To minimize coupling of Johnson noise from the metallic waveguide to the ground state of the V_B^- defects, an alumina spacer layer ($\sim$ 750 nm thick) was deposited on top of the gold waveguide, following established approaches.[1] A suitable hBN flake ($\sim$ 50 nm thick) was subsequently transferred onto the spacer layer. The microwave waveguide geometry was optimized using COMSOL simulations to maximize coupling between the microwave field and the V_B^- defects, while maintaining sufficient separation from the metal. Microwave pulses were generated using a gated microwave switch.

Flake Characterization

Figure S1 presents the photoluminescence (PL) intensity as a function of external magnetic field (H_{ext}) for the hBN flake containing uniformly implanted V_B^- defect centers, measured at temperatures of $T = 22, 115$, and 225 K. Pronounced PL dips are observed at $H_{ext} \sim \pm 0.075$ T and ± 0.125 T, corresponding to level anti-crossings in the excited and ground states, respectively.[2] In our experiments, we focus on magnetic fields $H_{ext} < 0.05$ T, $H_{ext} > 0.17$ T, to avoid the level anti-crossings, where the spin dynamics become more complex and out of the scope of this work.

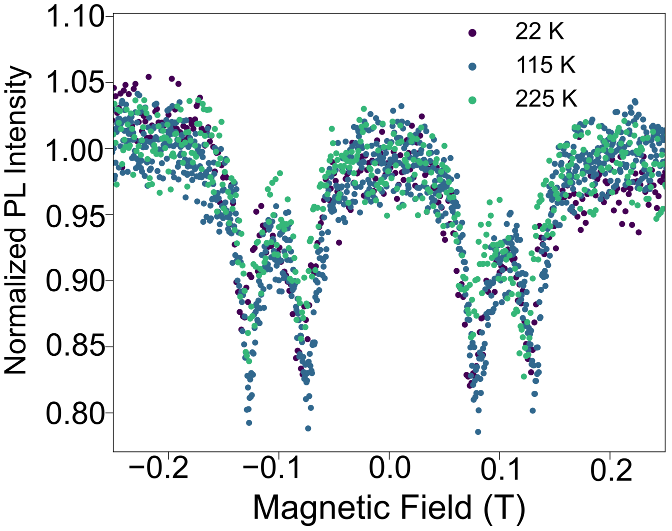

Figure S1: Normalized Photoluminescence signal (PL) strength vs magnetic field.

Figure S2 presents the atomic force microscopy (AFM) image of the hBN flake employed in this work for the measurements presented in the main text. The flake exhibits a uniform thickness of ~ 42 nm.

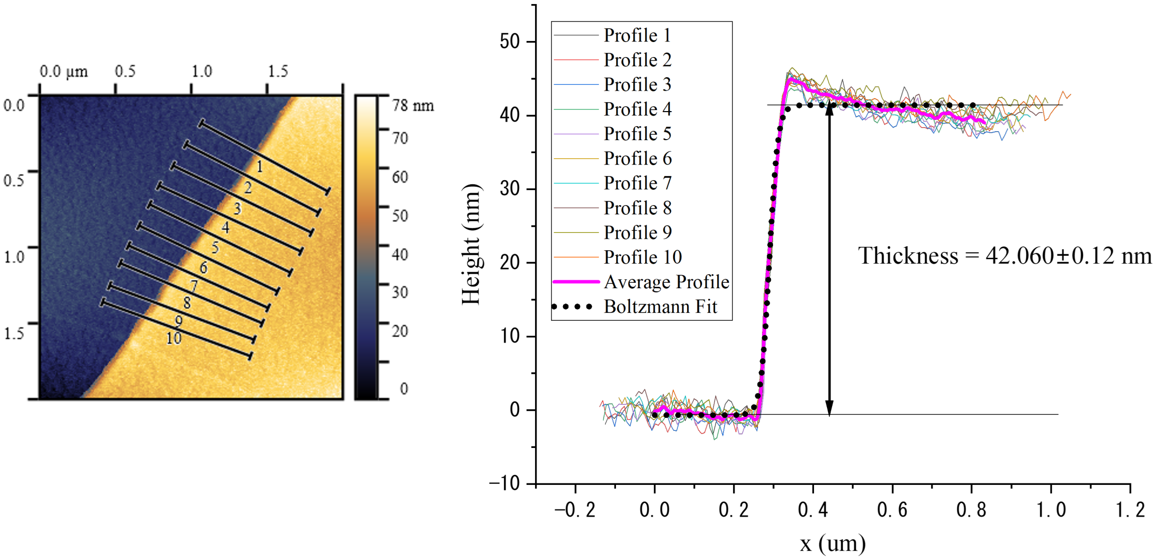

Figure S2: Atomic Force Microscopy image of the hBN flake on SiO_2/Si substrate. The average thickness of the flake is ~ 42 nm.

While helium ion implantation is an effective method for generating V_B^- defects, it can also introduce additional lattice disorder and background vacancies that may influence spin relaxation dynamics.[3] We study the extent of implantation-induced damage via Raman spectroscopy. Figure S3 shows the Raman spectrum of the hBN flake containing uniformly implanted V_B^- defect centers, measured under ambient conditions using a commercial Raman imaging microscope (Thermo Scientific DXR3xi) with a 532 nm excitation source and a $50x$ objective. The spectrum is well described by two Lorentzian peaks in the range 1100–1500 cm^{-1}. The higher-energy E_{2g} mode at $\sim$ 1380 cm^{-1} is intrinsic to hBN and remains unaffected by implantation. In contrast, the lower-energy D_1 peak at $\sim$ 1300 cm^{-1} emerges upon implantation and is attributed to V_B^- centers, with its intensity reflecting the defect density.

The density of negatively charged Boron-Vacancy center (V_B^-) in our sample is estimated to be $\sim$ 150 ppm, based on previous measurements using advanced dynamical decoupling techniques on a sample implanted under identical conditions.[4] Raman Spectroscopy provides an independent method to estimate the defect density. Following the approach of,[5] we estimate the density of V_B^- defects $\sim 10^{19} cm^{-3} (\simeq 100$ ppm) by measuring the ratio of the area under the curve of the implantation induced the lower-energy D_1 peak to the area under the curve of the E_{2g} peak ($A_{D_1}/A_{E_{2g}}$). Assuming a laser spot size $\sim 1 \mu m$ and the flake thickness $\sim$ 42 nm, the number of V_B^- defects measured in our scheme is $\sim 5 \times 10^5$.

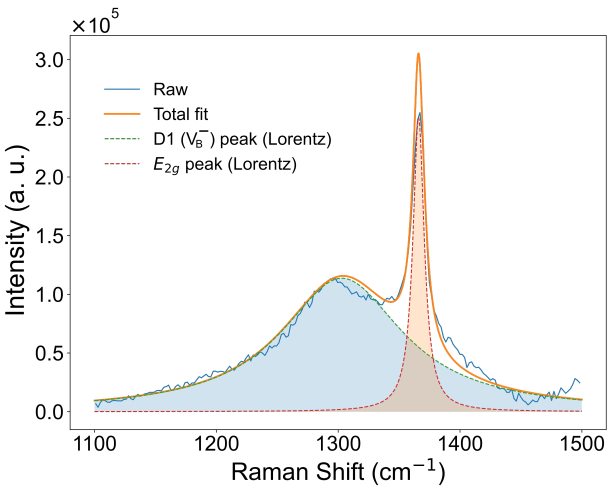

Figure S3: Raman scattering measurement of the hBN flake employed in this work with uniformly implanted V_B^- centers.

We also note that implantation induced disorder and unintended defect species may contribute to the formation of a fluctuating magnetic environment, particularly at low magnetic fields, where dipolar interactions play a more prominent role in the observed relaxation dynamics. A systematic investigation of the relationship between implantation dose, lattice disorder, defect charge-state conversion efficiency, and spin relaxation would require controlled variation of irradiation conditions and is beyond the scope of the present work.

Figure S4 presents the photoluminescence spectrum of V_B^- centers in hBN measured at temperature $T = 3$ K with a 700 nm long-pass filter.

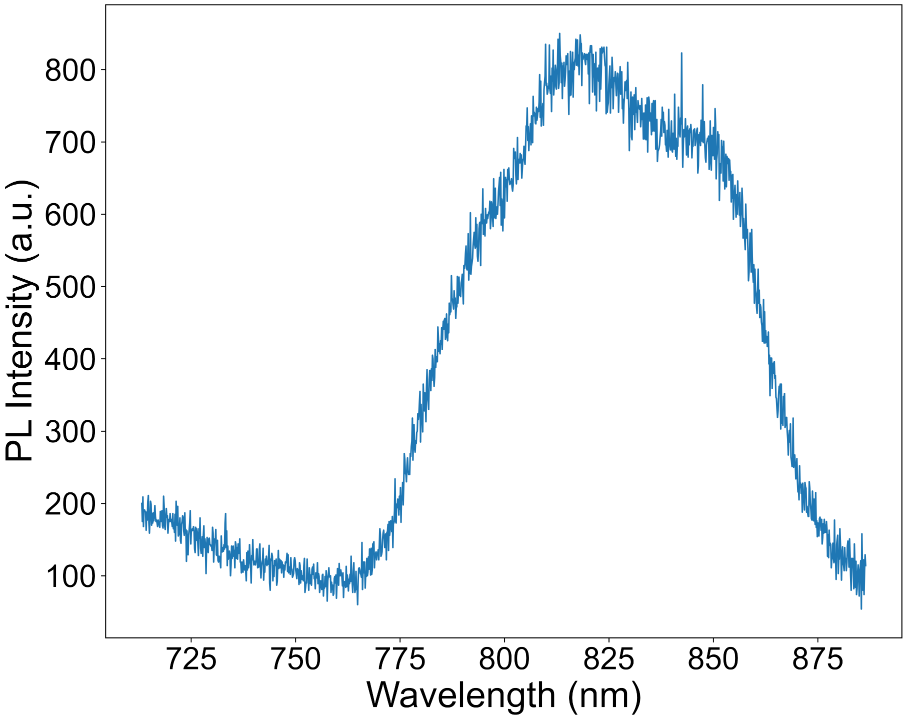

Figure S4: Photoluminescence spectrum of V_B^- centers in hBN at temperature $T = 3$ K.

Analytical fits of the experimental relaxation data with a stretched exponential

As mentioned in the main text, the spin-contrast as a function of delay time (Δt) is well described by a stretched exponential of the form $C_0(1 - \exp(-(\frac{\Delta t}{T_1})^\beta))$.[6] Here, C_0 is the spin-contrast amplitude, T_1 is the spin relaxation time, and β is the stretching factor. These parameters are extracted by fitting the experimental data at various temperatures and magnetic fields. Representative fits are shown in Figure S5: (a,b) $H_{ext} = 0.03, 0.04$ T at $T = 30$ K; (c) $H_{ext} = 0.03$ T at $T = 150$ K; and (d) $H_{ext} = 0.03$ T at $T = 250$ K. For comparison, fits using fixed stretching exponents of $\beta = 0.5$ and $\beta = 1$ are also included. The deviation of these fixed-exponent fits from the experimental data underscores the importance of treating β as a free parameter alongside $\Gamma = \frac{1}{T_1}$ during the fitting process.

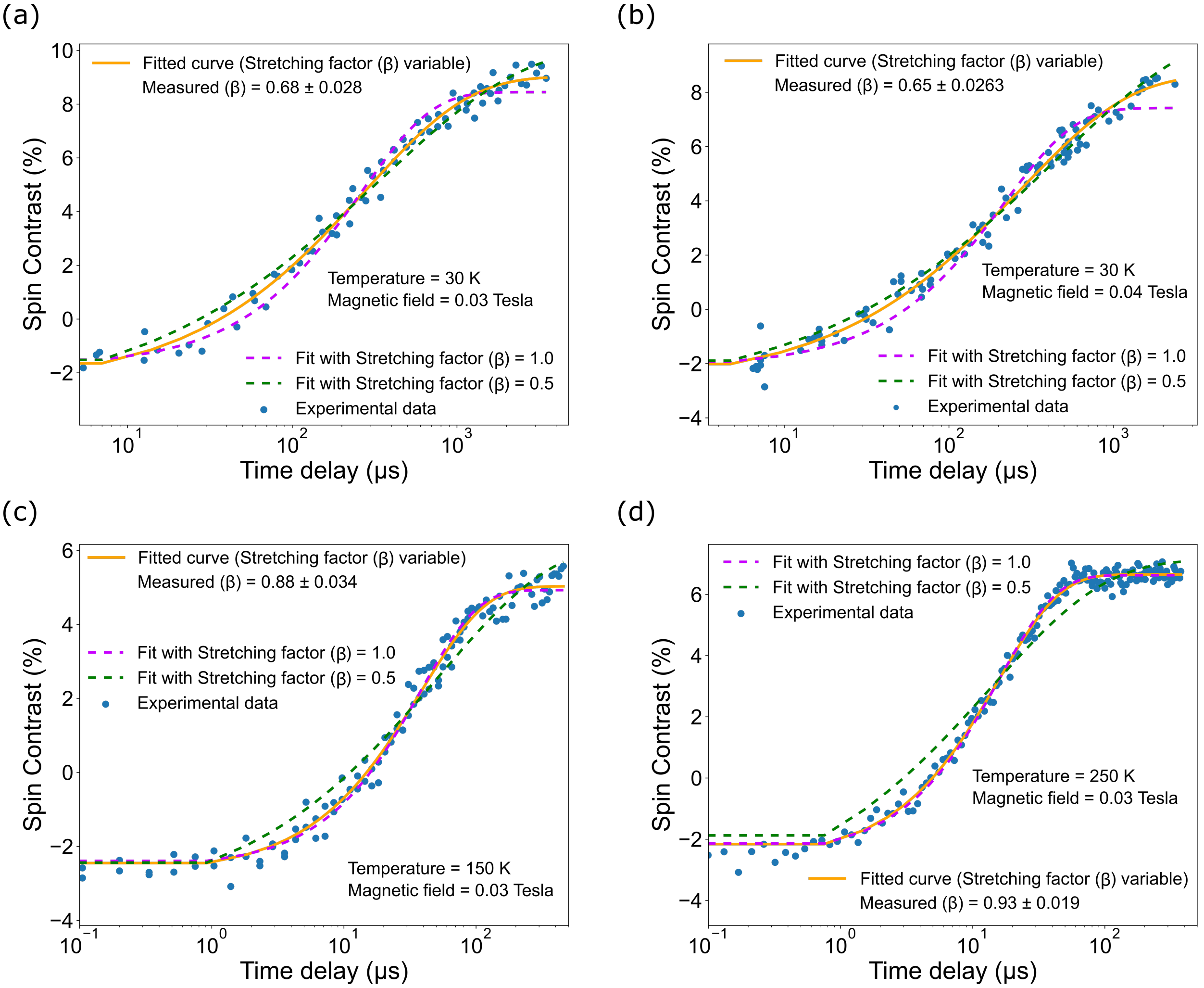

Figure S5: Measured T_1 relaxation curves (blue dots) of V_B^- centers in hBN at selected magnetic fields and temperatures, overlaid with fits to a stretched-exponential relaxation model (solid orange line). Additional fits with stretching factors fixed at $\beta = 1$ and $\beta = 0.5$ are included for comparison. (a, b) Magnetic field $= 0.03, 0.04$ T and Temperature $T = 30$ K. (c) Magnetic field $= 0.03$ T and Temperature $T = 150$ K. (d) Magnetic field $= 0.03$ T and Temperature $T = 250$ K.

To further investigate the origin of the stretched exponential decay behavior, we performed laser-power-dependent measurements for excitation powers over the range 0.8 to 4.0 mW. The experimental dataset, overlaid with stretched exponential fits, is presented in Figure S6. The measurement does not reveal a significant dependence of the measured relaxation rate or the stretching factor on the excitation power. On the other hand, the measured spin-contrast (C_0) exhibits a clear power dependence as illustrated in Figure S6. Across all

measured powers, a consistent offset of -2.5% is present. While the precise origin of this offset, whether arising from charge dynamics or experimental artifacts, remains uncertain, its magnitude is considerably smaller than the measured spin-contrast ($C_0 > 10\%$). It is not expected to affect the extracted parameters significantly.

To further evaluate the robustness of the fitting procedure, we refitted the experimental data after intentionally excluding data corresponding to dark time values (Δt) below a cutoff time. We present the result of this analysis for temperatures $T = 30, 50$ K in Figure S7 and Figure S8 respectively. In panels (a,b) of both figures, the experimental relaxation curves measured at $H_{ext} = 0.03, 7$ T are overlaid with fits obtained by systematically varying the cutoff time between 0.01 to 20 μs. Panels (c,d) display the corresponding magnetic-field dependence of the extracted relaxation rate Γ and stretching factor β as a function of the cutoff time. No systematic dependence on the cutoff time is observed, aside from isolated deviations at a few points. These results reinforce the robustness of the fitting approach and confirm that the initial negative offset does not significantly affect the extracted parameters.

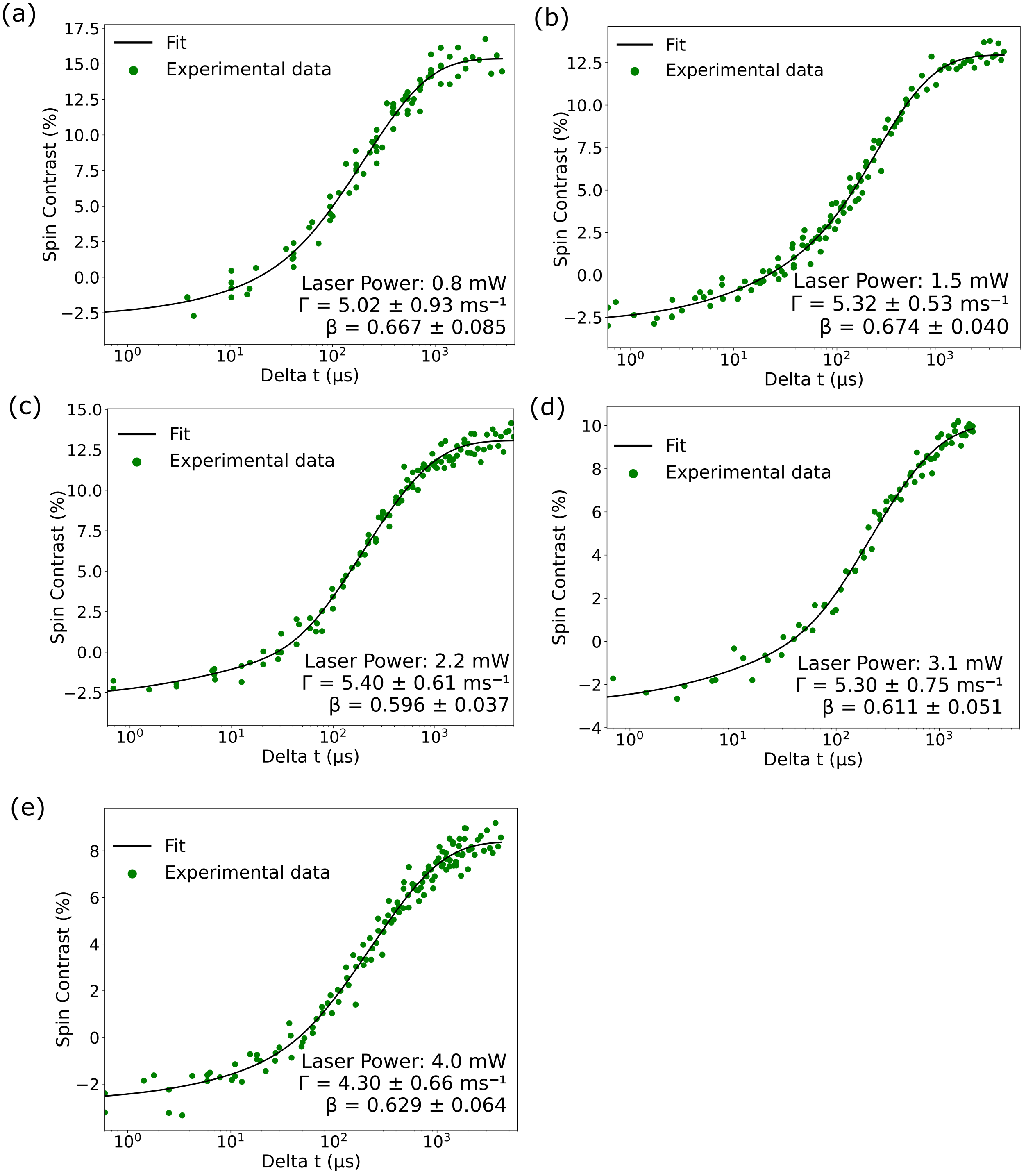

Figure S6: Laser power dependence of the relaxation rate (Γ) and stretching factor (β) at temperature $T = 30$ K and magnetic field $H_{ext} = 0.02$ T. The observed variation in the measured relaxation rate and stretching factor lie within the fitting uncertainties. Panels (a-e) correspond to laser powers of 0.8, 1.5, 2.2, 3.1, 4.0 mW, respectively.

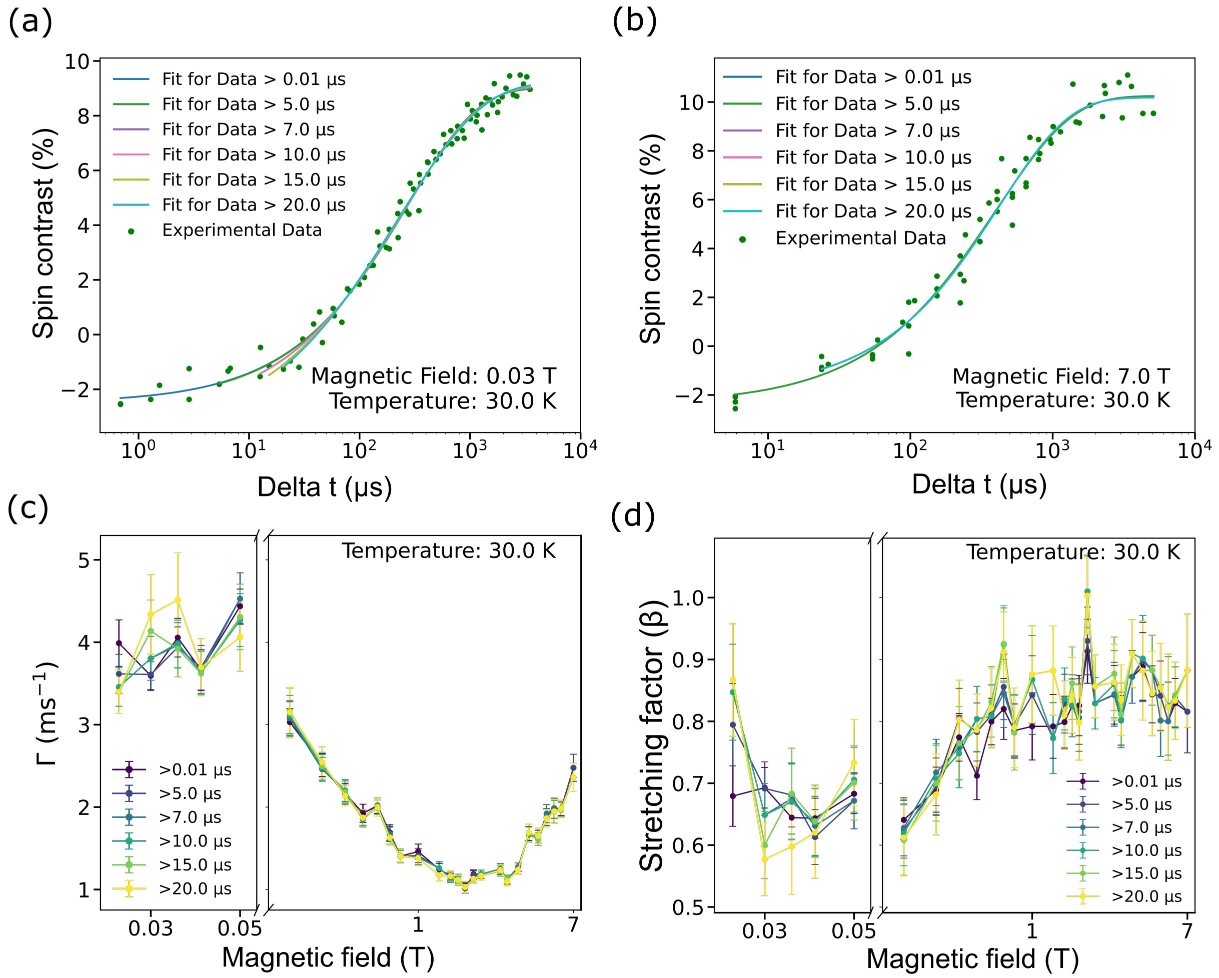

Figure S7: Robustness of the stretched-exponential fitting procedure at $T = 30$ K. Panels (a,b) show representative relaxation curves measured at $H_{ext} = 0.03$ and 7 T, overlaid with fits obtained by excluding data below cutoff times ranging from 0.01 to 20 μs. Panels (c,d) display the extracted relaxation rate Γ and stretching exponent β as a function of magnetic field for the same range of cutoff times. No systematic dependence on the cutoff is observed, aside from isolated deviations at a few points.

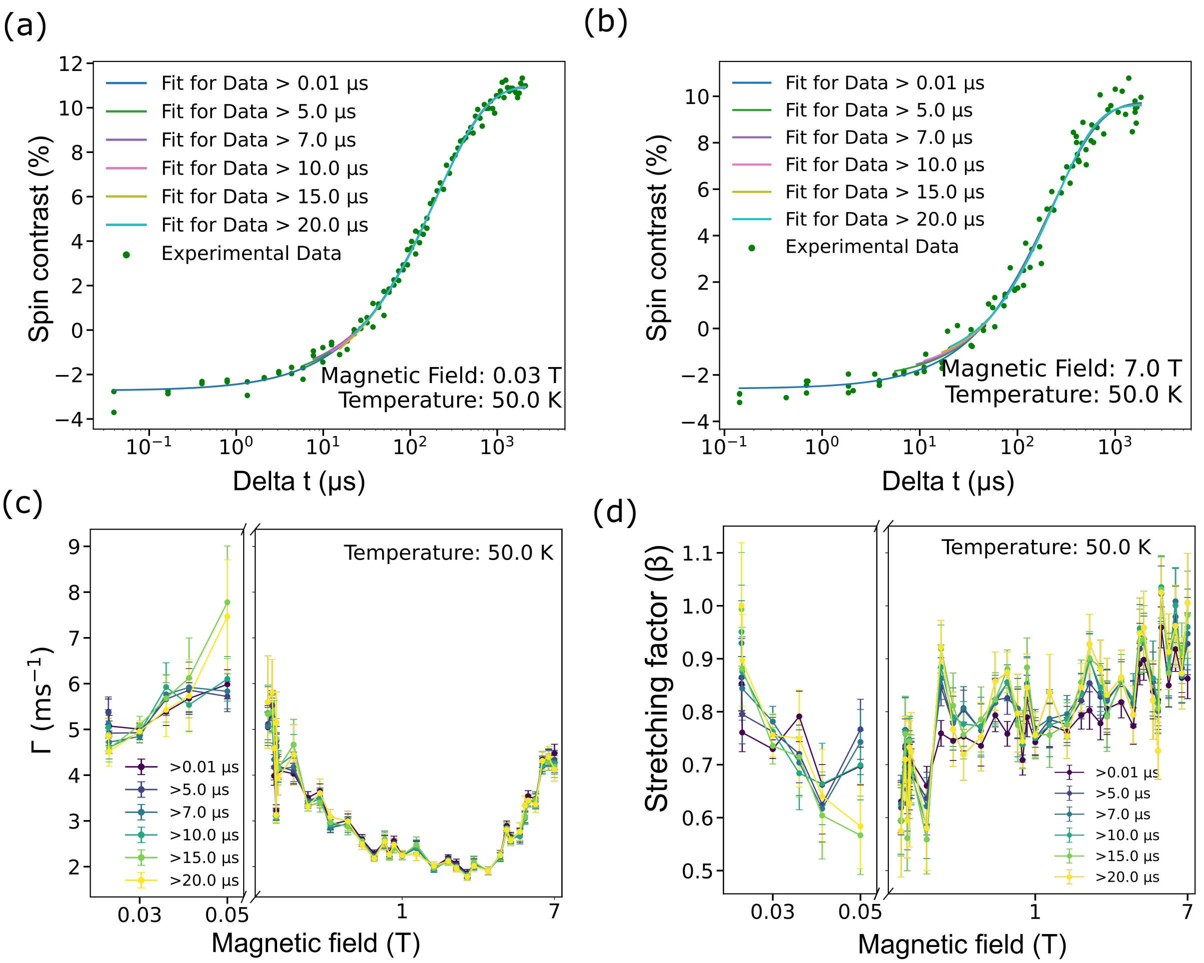

Figure S8: Robustness of the stretched-exponential fitting procedure at $T = 50$ K. Panels (a,b) show representative relaxation curves measured at $H_{ext} = 0.03$ and 7 T, overlaid with fits obtained by excluding data below cutoff times ranging from 0.01 to 20 μs. Panels (c,d) display the extracted relaxation rate Γ and stretching exponent β as a function of magnetic field for the same range of cutoff times. No systematic dependence on the cutoff is observed, aside from isolated deviations at a few points.

Microwave-assisted differential measurements

In our experiments, we use 10 μs pulses of 532 nm laser to initialize and readout of the ground-state spin population distribution of the defects. Initialization relies on spin-dependent intersystem crossing (ISC) that polarizes the ground state into the $m_s = 0$ spin sublevel. A variable dark interval (Δt) then allows the system to relax toward thermal equilibrium, after which a second pulse reads out the population via the same spin-dependent pathway.

Ideally, the laser pulses would only modify the population distribution within the ground-state spin manifold. However, the excitation laser pulse can also alter the population of the negatively charged boron vacancy (V_B^-) centers, via photo-ionization and recombination processes, as observed for NV centers in diamond.[7,8] This transient charge distribution also relaxes towards the steady-state charge distribution during the dark interval. The transient dynamics of V_B^- population modulates the PL signal strength. Consequently, the measured relaxation profile reflects a sum of both spin- and charge-relaxation processes.

$$S(\Delta t) = A_s\left[1 - e^{-(\Delta t/T_1)^\beta}\right] + A_c\left[1 - e^{-\Delta t/T_c}\right] + C\,. \tag{1}$$

Parameters.

- Δt: dark interval between pump and readout pulses.
- A_s: spin-contrast amplitude.
- T_1: longitudinal spin-relaxation time.
- $\beta \in (0, 1]$: stretching exponent related to spin-relaxation
- A_c: amplitude due to charge re-equilibration (its sign encodes whether PL rises or falls).
- T_c: charge re-equilibration time constant.
- C: baseline offset (e.g., residual background).

The spin-relaxation parameters (A_s, T_1) are expected to exhibit temperature and magnetic field dependence due to spin-phonon and spin-spin interaction,[9] whereas transient charge-dynamics parameters (A_c, T_c) should exhibit a much stronger dependence on the wavelength of the excitation laser and excitation power.[6] If the charge-dynamics rate $(1/T_c)$ is indeed independent of temperature and magnetic field, it would manifest as a constant offset in the measured spin-relaxation rates. However, the presence of multiple exponential

processes can effectively mimic a stretched exponential profile. Furthermore, the extent of charge dynamics could have a spatial variation, which could further contribute to the observed stretched-exponential profile. To disentangle these different processes, we employ a microwave-assisted differential measurement scheme designed to eliminate transient charge-related dynamics and recover the intrinsic spin-relaxation behavior. Since coplanar waveguides cannot be used to coherently drive the V_B^- centers at Tesla-scale magnetic fields, due to ground state splitting > 20 GHz, we restrict the measurements to external magnetic fields of a few hundred Gauss.

This modified measurement scheme is illustrated in Figure S9(a). The first green pulse initializes the ground state into the $m_s = 0$ state. After a variable dark interval, a second green pulse is applied to read out the ground-state population. The number of photons collected in a short time window at the onset of this pulse (N_1) provides a measure of the time-dependent spin population. Toward the end of the same pulse, the spin is reinitialized into the $m_s = 0$ state, followed by another dark interval of equal duration. A resonant π-pulse (duration ~ 10 ns) is then applied before a third green pulse is used for readout. The photons detected in the corresponding short interval at the beginning of this pulse yield N_2, which reflects the spin population after the π-pulse. The number of photons measured at the beginning of the first green pulse serves as a reference signal (N_0) corresponding to the ground state spin initialized into the $m_s = 0$ state.

The bright curve, defined as $\frac{N_0 - N_1}{N_0}$, corresponds to the microwave-free all-optical relaxation signal used for the measurements presented in the main text. The dark curve is measured as $\frac{N_0 - N_2}{N_0}$ and the differential curve is measured as $\frac{N_2 - N_1}{N_0}$. The dark curve carries the same laser pulse-induced charge dynamics contribution as the bright curve ($A_c\left[1 - e^{-\Delta t / T_c}\right]$), which is effectively canceled out in the differential scheme.

In Figure S9(b,c,d) we present the measurements performed at temperatures $T = 8, 12$ and 16 K, respectively. The results show that the differential curves also exhibit a stretched exponential decay profile. Interestingly, the stretching in the differential curve is even more

pronounced than in the bright curve. The differential scheme yields a relaxation rate Γ that is marginally higher than the value obtained from the bright curve; however, the increased fitting error reflects the smaller signal contrast inherent to this approach. This observation suggests that transient charge dynamics make only a minor contribution in the all-optical measurement scheme, which would otherwise result in a reduced rate in the differential scheme.

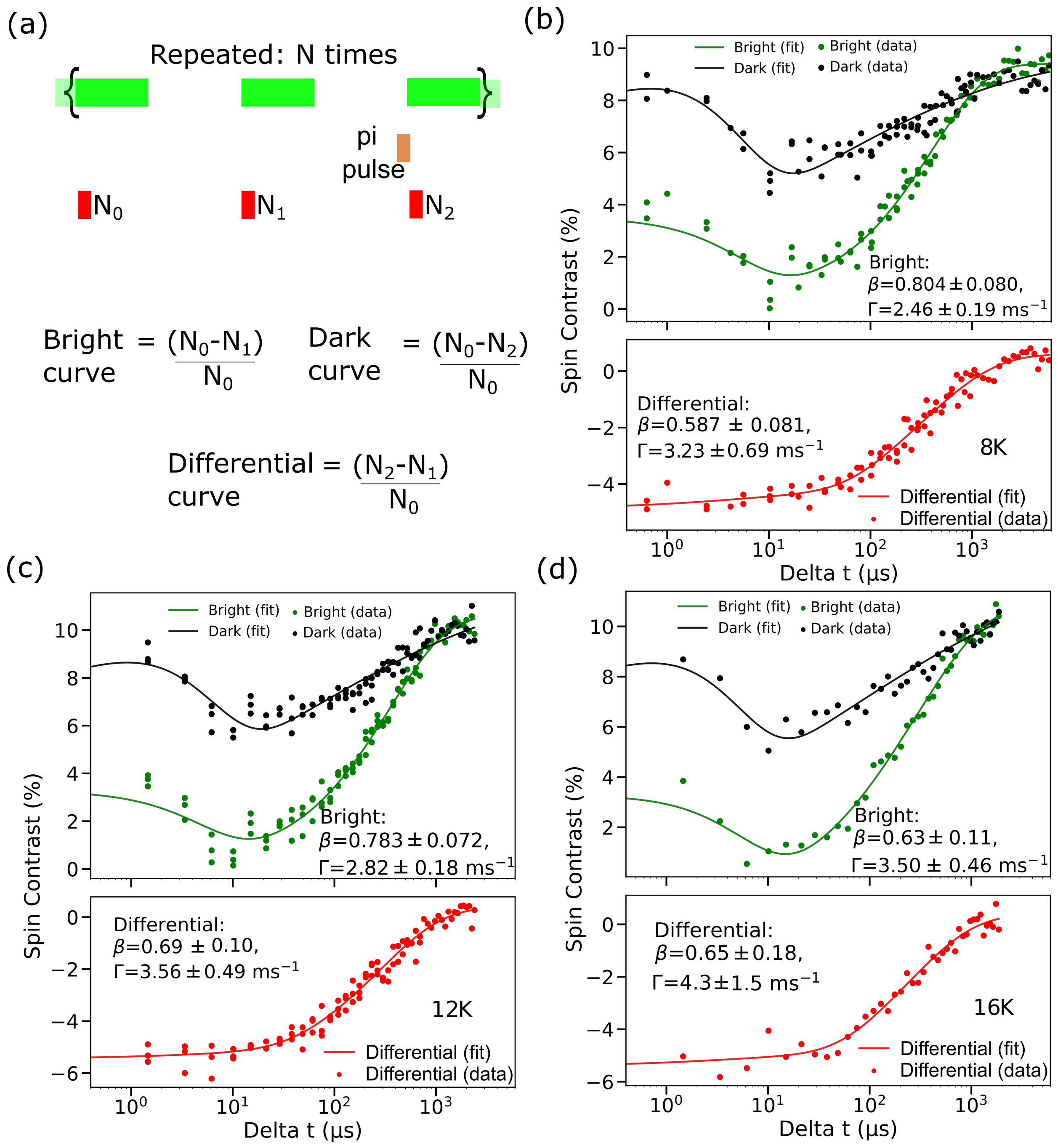

Figure S9: Microwave-assisted differential measurement scheme to identify charge-dynamics contributions. (a) Schematic of the differential protocol, where alternating microwave on/off sequences are used to isolate spin-related relaxation signals from background charge effects. (b–d) Representative relaxation curves measured at temperature $T = 8, 12, 16$ K, shown with microwave-on (green), microwave-off (black) and differential (red) traces overlaid with fits.

In addition to these observations, we note that the significant magnetic field dependence

of the relaxation rate presented in the main text naturally rules out the possibility of charge dynamics dominating the measurement scheme. Intuitively, we expect the extent of charge dynamics to be dependent on the energy levels of the conduction band and valence band of electrons relative to the energy levels of the negatively-charged and neutral V_B^- centers. These energy scales would determine the ionization rate for a given wavelength of excitation laser. However, these rates are not affected by the magnetic field, which only leads to a change in the ground state spin splitting.

We also note that the differential scheme is only effective at rejecting the pulse-synchronous, laser-induced charge dynamics which result in a non-equilibrium charge distribution. As discussed above, this transient charge state relaxes towards a steady-state charge state which could result in an additional exponential curve ($A_c\big[1 - e^{-\Delta t/T_c}\big]$). This additional exponential is rejected in microwave assisted differential scheme. However, it doesn't rule out the possibility of steady-state charge dynamics after the charge state population has stabilized during the dark interval ($\Delta t >> T_c$) in the measurement scheme. In a real system, steady-state population exchange between different charge species is expected to occur ($V_B^- \Leftrightarrow V_B^0$). In this scenario, the population of V_B^- is fixed, resulting in a fixed PL signal. But the charge dynamics would result in a loss of spin information. Therefore, charge dynamics could manifest as an additional channel for spin-relaxation. These complex factors can be disentangled by a systematic investigation of the relaxation rate for different implantation dosages and different excitation laser wavelengths, which is beyond the scope of this work.

Data analysis and fitting

As outlined in the Phenomenological Description section of the main text, we model the magnetic-field and temperature dependence of the relaxation rate using a combined analytical framework.

The spin–phonon contribution is described by

$$\Gamma^{\text{spin-ph}} = A_1 T \omega_0^{n_1} + A_2 T^{n_2}, \tag{2}$$

where A_1 and A_2 are coupling constants, T is the sample temperature, and n_1 and n_2 are scaling exponents.[9–13]

The remaining contribution is captured by a Lorentzian form,

$$\Gamma^{\text{Lorentzian}} = \gamma^2 S(\omega_0) = \frac{\eta \tau_c}{1 + (\omega_0 \tau_c)^2}, \tag{3}$$

where γ is the gyromagnetic ratio, $\eta(T)$ is a phenomenological parameter reflecting the density and dipolar coupling of fluctuating spins, and $\tau_c(T)$ is the correlation time. Here, $\eta(T)$ and $\tau_c(T)$ serve as fitting parameters, providing quantitative insight into the properties of the Lorentzian bath. The ground-state spin splitting between $m_s = 0 \leftrightarrow m_s = \pm 1$ transitions is represented as ω_0. In the fitting procedure, we take both the transitions into account.

The total relaxation rate is then expressed as a sum of the two contributions,

$$\Gamma(H_{ext}, T) = \Gamma^{\text{Lorentzian}} + \Gamma^{\text{spin-ph}}, \tag{4}$$

which allows us to extract the relative contributions of the different relaxation mechanisms.

The V_B^- defects form a $S = 1$ ground-state spin manifold, where the $m_s = +1$ and $m_s = -1$ states are not strictly degenerate. Consequently, the spin levels constitute an effective three-level system that must be considered when interpreting and fitting the relaxation dynamics. In our scheme, the measured relaxation rate ($\Gamma(H_{ext}, T)$) is obtained from the decay of the $m_s = 0$ population, includes contributions from transitions $m_s = 0 \leftrightarrow m_s = \pm 1$ as well as the $m_s = +1 \leftrightarrow m_s = -1$.[10]

For a qualitative understanding of the results, we consider the following different regimes of magnetic field and temperature as mentioned in the main text. In the low-field regime

($H_{ext} < 0.05$ T), the difference between the $m_s = 0 \leftrightarrow m_s = +1$ and $m_s = 0 \leftrightarrow m_s = -1$ transitions is small compared to the fluctuation rate of the Lorentzian bath ($1/\tau_c$). In the intermediate-field regime (0.17 T $< H_{ext} <$ 1.8 T), the absolute transition energies increase, however, their separation remains small relative to the overall spectral width of the Lorentzian bath. At higher magnetic fields ($H_{ext} >$ 1.8 T), relaxation is dominated by first-order spin–phonon processes that are sensitive to the absolute transition frequencies. However, the relative difference between the $m_s = 0 \leftrightarrow m_s = +1$ and $m_s = 0 \leftrightarrow m_s = -1$ transition energies remains small compared to their absolute values, and both transitions couple to a continuum of acoustic phonons.

An important distinction between the two processes is that the double quantum transition between $m_s = +1 \leftrightarrow m_s = -1$ spin-levels can be driven by spin–phonon coupling, whereas such a process is not permitted through spin–spin interactions.[10] In our experiments, the splitting between the $m_s = +1 \leftrightarrow m_s = -1$ states reaches as high as 392 GHz at $H_{ext} = 7$ T, which can have important implications for the first-order spin-phonon coupling. Therefore, the most general form of spin-phonon contribution can be expressed as

$$\Gamma^{\text{spin-ph}} = A_1 T(\frac{D + g\mu_B H_{ext}}{\hbar})^{n_1} + A_1 T(\frac{D - g\mu_B H_{ext}}{\hbar})^{n_1} + A_1 T(\frac{2g\mu_B H_{ext}}{\hbar})^{n_1} + A_2 T^{n_2}, \quad (5)$$

In this expression, we assume that the coupling constant A_1 and the exponent for the first-order spin phonon coupling n_1 remain the same for the $m_s = 0 \leftrightarrow m_s = \pm 1$ and $m_s = +1 \leftrightarrow m_s = -1$ transitions.[10] This assumption can be tested by first-principles calculations and further measurements. For $H_{ext} >> \frac{D}{g\mu_B}$, where the first-order term is expected to be crucial, and $\hbar\omega_0 < k_B T$, the first-order spin-phonon coupling is $\sim T(H_{ext})^{n_1}$.

To ensure consistency between the temperature- and field-dependent analyses, we employ an iterative fitting approach. We begin by extracting the exponent n_1 by fitting the magnetic-field–dependent dataset at several fixed temperatures ($T = 30, 50, 100, 150, 250$ K), for a

fixed value of $n_2 \sim 2$ based on prior measurements of second-order spin-phonon temperature-dependence[13]). This step allows us quantitatively determine the key fitting parameters using the combined model described in Equation 4. In this framework, we extract global spin-phonon coupling parameters A_1, A_2, n_1, while allowing the Lorentzian bath parameters to vary independently with temperature $\eta(T), \tau_c(T)$. Following this, we use the extracted value of n_1 as a fixed-parameter to fit the temperature-dependent dataset for several magnetic fields ($H_{ext} = 0.03, 0.04, 1.65, 1.8, 2.0, 6.0, 7.0$ T) and determine a global value of n_2. The updated value of n_2 is subsequently used as input for refining the magnetic-field fits. This process is repeated until the parameters converge, yielding a mutually consistent set of fit values for n_1 and n_2. The extracted fit parameters as a function of temperature from the magnetic-field dependent fits are presented in Table 1. The extracted fit parameters as a function of magnetic-field from the temperature-dependent fits are presented in Table 2.

Table 1: Extracted fitting parameters as a function of temperature for magnetic field dependent fits.

Temperature (K)	A_1 ($[ms]^{-1}K^{-1}(rad/s)^{-n_1}$)	A_2 ($[ms]^{-1}K^{-n_2}$)	n_1	n_2	η ($[ms]^{-2}$)	τ_c (ps)
30	$(1.95 \pm 1.15) \times 10^{-21}$	$(3.19 \pm 0.02) \times 10^{-4}$	1.56 ± 0.05	2.16	$(1.54 \pm 0.04) \times 10^{8}$	9.4 ± 0.4
50	$(1.95 \pm 1.15) \times 10^{-21}$	$(3.19 \pm 0.02) \times 10^{-4}$	1.56 ± 0.05	2.16	$(1.16 \pm 0.03) \times 10^{8}$	15.8 ± 0.7
100	$(1.95 \pm 1.15) \times 10^{-21}$	$(3.19 \pm 0.02) \times 10^{-4}$	1.56 ± 0.05	2.16	$(2.51 \pm 0.09) \times 10^{8}$	12.5 ± 0.7
150	$(1.95 \pm 1.15) \times 10^{-21}$	$(3.19 \pm 0.02) \times 10^{-4}$	1.56 ± 0.05	2.16	$(5.13 \pm 0.21) \times 10^{8}$	9.8 ± 0.5
250	$(1.95 \pm 1.15) \times 10^{-21}$	$(3.19 \pm 0.02) \times 10^{-4}$	1.56 ± 0.05	2.16	$(6.18 \pm 0.44) \times 10^{8}$	13.7 ± 1.4

Table 2: Extracted fitting parameters as a function of magnetic field for the temperature-dependent fits.

B_{field} (T)	A_1 ($[ms]^{-1}K^{-1}(rad/s)^{-n_1}$)	A_2 ($[ms]^{-1}K^{-n_2}$)	n_1	n_2	η ($[ms]^{-2}$)	τ_c (ps)
0.03	$(4.2 \pm 0.07) \times 10^{-21}$	$(3.24 \pm 0.22) \times 10^{-4}$	1.545	2.17 ± 0.01	$(1.80165 \pm 0.00003) \times 10^{8}$	12.35
0.04	$(4.2 \pm 0.07) \times 10^{-21}$	$(3.24 \pm 0.22) \times 10^{-4}$	1.545	2.17 ± 0.01	$(1.80165 \pm 0.00003) \times 10^{8}$	12.35
1.65	$(4.2 \pm 0.07) \times 10^{-21}$	$(3.24 \pm 0.22) \times 10^{-4}$	1.545	2.17 ± 0.01	$(1.80165 \pm 0.00003) \times 10^{8}$	12.35
1.8	$(4.2 \pm 0.07) \times 10^{-21}$	$(3.24 \pm 0.22) \times 10^{-4}$	1.545	2.17 ± 0.01	$(1.80165 \pm 0.00003) \times 10^{8}$	12.35
2.0	$(4.2 \pm 0.07) \times 10^{-21}$	$(3.24 \pm 0.22) \times 10^{-4}$	1.545	2.17 ± 0.01	$(1.80165 \pm 0.00003) \times 10^{8}$	12.35
6.0	$(4.2 \pm 0.07) \times 10^{-21}$	$(3.24 \pm 0.22) \times 10^{-4}$	1.545	2.17 ± 0.01	$(1.80165 \pm 0.00003) \times 10^{8}$	12.35
7.0	$(4.2 \pm 0.07) \times 10^{-21}$	$(3.24 \pm 0.22) \times 10^{-4}$	1.545	2.17 ± 0.01	$(1.80165 \pm 0.00003) \times 10^{8}$	12.35

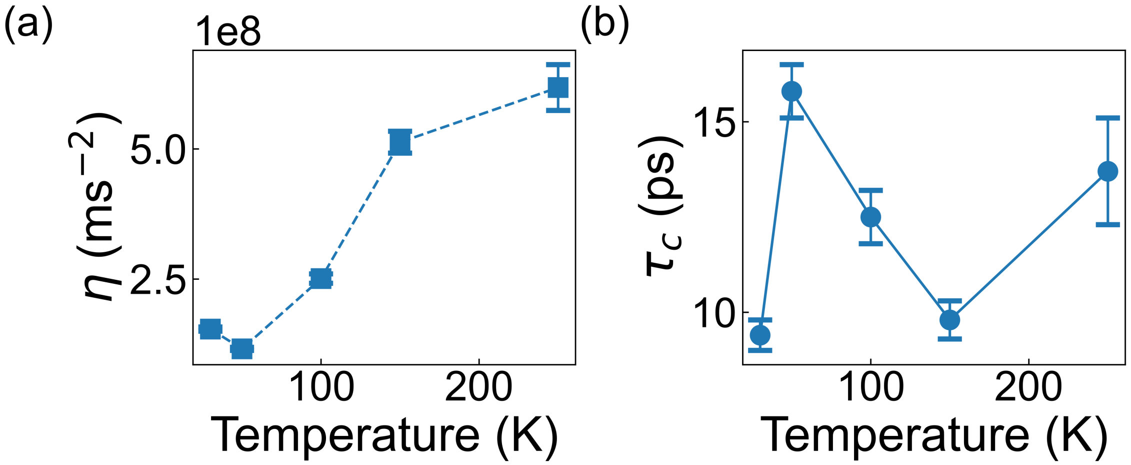

Figure S10: (a) Coupling strength of the Lorentzian bath ($\eta(T)$) vs Temperature (b) Correlation time ($\tau_c(T)$) of the Lorentzian bath vs Temperature.

Several observations are in order. When we fit the magnetic-field dependent dataset, the parameter $\eta(T)$ exhibits an approximately linear dependence on temperature in the range 250–50 K, followed by a tendency to saturate at lower temperatures. In contrast, the correlation time $\tau_c(T)$ does not display a clear systematic dependence on temperature within the experimental uncertainty. When fitting the temperature-dependent data, we assume that the Lorentzian bath parameters η and τ_c remain fixed, as their temperature-dependence is not known *a priori*. Notably, the extracted value of η from this procedure is found to be close to its low-temperature value obtained from the magnetic-field-dependent fits. This agreement supports our interpretation that the Lorentzian contribution is most prominent in the low-temperature regime, where it dominates the relaxation dynamics.

The parameters A_1, A_2, n_1, and n_2 are treated as global fit parameters in our phenomenological model. Any additional temperature-dependent structure associated with the microscopic spin–phonon coupling cannot be unambiguously resolved without detailed first-principles calculations.

First-principles calculation of the phonon spectrum

We performed first-principles calculations of the phonon spectrum of monolayer hBN containing a V_B^- center. The phonon band structure showed in Figure S11 was obtained using the Vienna *Ab initio* Simulation Package,[14] employing the projector augmented wave (PAW) method, with exchange correlation treated within the generalized gradient approximation using the Perdew–Burke–Ernzerhof functional.[15] The calculations were performed on a $6 \times 6 \times 1$ supercell of monolayer hBN with a vacuum spacing of 20 Å, consistent with previous studies.[16] A Γ-centered $4 \times 4 \times 1$ k-point mesh was used for these calculations. The plane-wave energy cutoff was set to 520 eV. Electronic self-consistency was achieved with an energy convergence threshold of 10^{-7} eV. Structural relaxations were carried out until the residual forces on all atoms were below 10^{-4} eV/Å. Following this, the phonon frequencies were calculated using the finite displacement method as implemented in PHONOPY package.[17]

These calculations provide insight into the relevant phonon modes and their energy scales, enabling us to distinguish between low-energy acoustic phonons that mediate first-order (resonant) spin–phonon processes and higher-energy optical phonons that contribute to second-order (Raman-like) relaxation mechanisms. Consistent with previous reports,[13,16] we identify phonon modes in the $\sim 5 - 20$ meV range as the dominant contributors to the second-order spin-phonon coupling. In contrast, the first-order spin-phonon process is mediated by resonant coupling to low-energy acoustic phonons that match the ground-state splitting (~ 0.2 THz at 7 T).

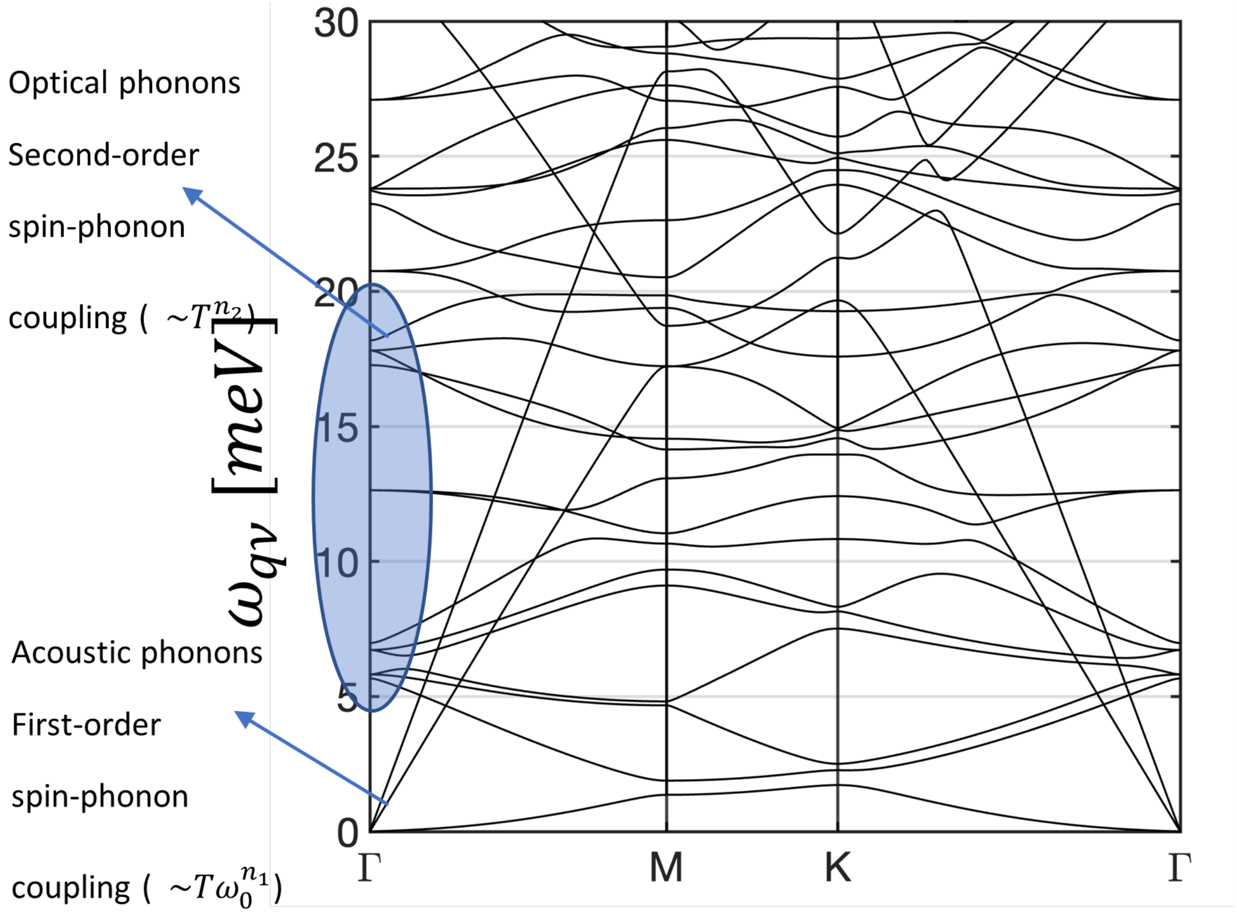

Figure S11: First-principles calculation of the phonon spectrum of monolayer hexagonal Boron Nitride with a single Boron-Vacancy Center (V_B^-). The shaded area represents the optical phonons that drive the second-order spin phonon coupling, whereas the low-energy acoustic phonons drive the first-order spin-phonon coupling.

Additional temperature-dependent data

Additional temperature-dependent data at fixed magnetic fields $H_{ext} = 0.04, 1.65, 2.0, 6.0$ T is presented in Figure S12.

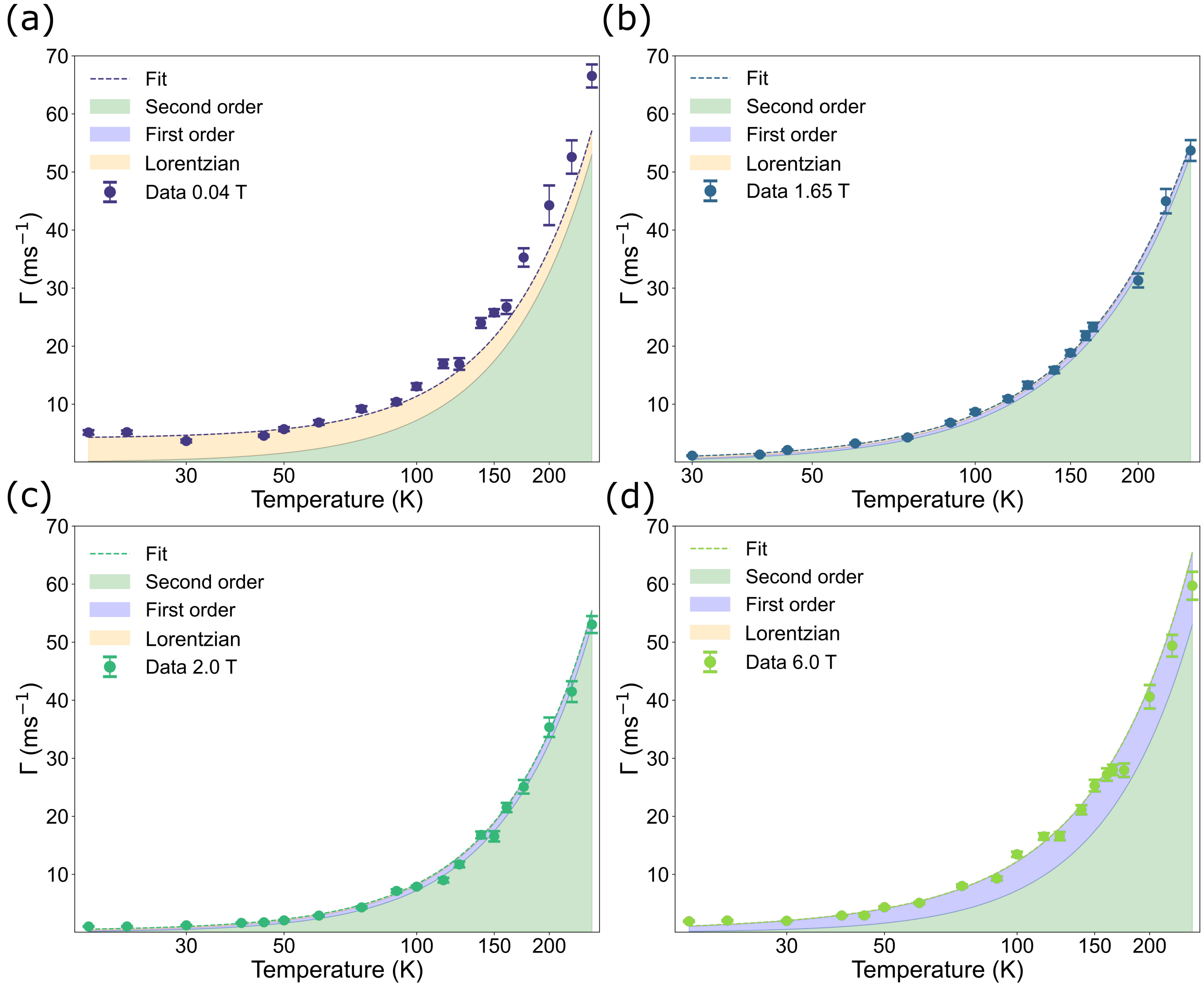

Figure S12: Temperature dependence of the relaxation rate ($\Gamma = 1/T_1$) at selected magnetic fields, showing the decomposition into individual contributions. Panels (a–d) correspond to $H_{ext} = 0.04, 1.65, 1.8, 6.0$ T, respectively.

References

(1) Kolkowitz, S.; Safira, A.; High, A. A.; Devlin, R. C.; Choi, S.; Unterreithmeier, Q. P.; Patterson, D.; Zibrov, A. S.; Manucharyan, V. E.; Park, H.; Lukin, M. D. Probing

Johnson noise and ballistic transport in normal metals with a single-spin qubit. *Science* **2015**, *347*, 1129–1132.

(2) Clua-Provost, T. et al. Spin-dependent photodynamics of boron-vacancy centers in hexagonal boron nitride. *Phys. Rev. B* **2024**, *110*, 014104.

(3) Vlassiouk, I. V.; Wu, Y.-C.; Puretzky, A.; Liang, L.; Lasseter, J.; Dryzhakov, B.; Gallagher, I.; Ghosh, S.; Lavrik, N.; Dyck, O.; others Defect Engineering in Large-Scale CVD-Grown Hexagonal Boron Nitride: Formation, Spectroscopy, and Spin Relaxation Dynamics. *arXiv preprint arXiv:2503.18894* **2025**,

(4) Gong, R.; He, G.; Gao, X.; Ju, P.; Liu, Z.; Ye, B.; Henriksen, E. A.; Li, T.; Zu, C. Coherent dynamics of strongly interacting electronic spin defects in hexagonal boron nitride. *Nature Communications* **2023**, *14*.

(5) Patra, A.; Konrad, P.; Sperlich, A.; Biktagirov, T.; Schmidt, W. G.; Spencer, L.; Aharonovich, I.; Höfling, S.; Dyakonov, V. Quantifying Spin Defect Density in hBN via Raman and Photoluminescence Analysis. *Advanced Functional Materials* **2025**, e17851.

(6) Choi, J.; Choi, S.; Kucsko, G.; Maurer, P.; Shields, B.; Sumiya, H.; Onoda, S.; Isoya, J.; Demler, E.; Jelezko, F.; Yao, N.; Lukin, M. Depolarization Dynamics in a Strongly Interacting Solid-State Spin Ensemble. *Physical Review Letters* **2017**, *118*, 093601.

(7) Giri, R.; Gorrini, F.; Dorigoni, C.; Avalos, C. E.; Cazzanelli, M.; Tambalo, S.; Bifone, A. Coupled charge and spin dynamics in high-density ensembles of nitrogen-vacancy centers in diamond. *Phys. Rev. B* **2018**, *98*, 045401.

(8) Cardoso Barbosa, I.; Gutsche, J.; Widera, A. Impact of charge conversion on NV-center relaxometry. *Phys. Rev. B* **2023**, *108*, 075411.

(9) Jarmola, A.; Acosta, V. M.; Jensen, K.; Chemerisov, S.; Budker, D. Temperature- and

Magnetic-Field-Dependent Longitudinal Spin Relaxation in Nitrogen-Vacancy Ensembles in Diamond. *Physical Review Research* **2012**, *108*, 197601.

(10) Norambuena, A.; Muñoz, E.; Dinani, H. T.; Jarmola, A.; Maletinsky, P.; Budker, D.; Maze, J. R. Spin-lattice relaxation of individual solid-state spins. *Physical Review B* **2018**, *97*, 094304.

(11) Cambria, M. C.; Gardill, A.; Li, Y.; Norambuena, A.; Maze, J. R.; Kolkowitz, S. State-dependent phonon-limited spin relaxation of nitrogen-vacancy centers. *Physical Review Research* **2021**, *3*, 013123.

(12) Cambria, M. C.; Norambuena, A.; Dinani, H. T.; Thiering, G.; Gardill, A.; Kemeny, I.; Li, Y.; Lordi, V.; Gali, Á.; Maze, J. R.; Kolkowitz, S. Temperature-Dependent Spin-Lattice Relaxation of the Nitrogen-Vacancy Spin Triplet in Diamond. *Physical Review Letters* **2023**, *130*, 256903.

(13) Liu, Z.; Gong, R.; Huang, B.; Jin, Y.; Du, X.; He, G.; Janzen, E.; Yang, L.; Henriksen, E. A.; Edgar, J. H.; Galli, G.; Zu, C. Temperature-dependent spin-phonon coupling of boron-vacancy centers in hexagonal boron nitride. *Phys. Rev. B* **2025**, *111*, 024108.

(14) Kresse, G.; Furthmüller, J. Efficient iterative schemes for ab initio total-energy calculations using a plane-wave basis set. *Phys. Rev. B* **1996**, *54*, 11169–11186.

(15) Perdew, J. P.; Burke, K.; Ernzerhof, M. Generalized Gradient Approximation Made Simple. *Phys. Rev. Lett.* **1996**, *77*, 3865–3868.

(16) Estaji, N.; Sarsari, I. A.; Thiering, G. m. H.; Gali, A. Spin-phonon relaxation of boron vacancy centers in two-dimensional boron nitride polytypes. *Phys. Rev. B* **2025**, *112*, L201407.

(17) Togo, A.; Tanaka, I. First principles phonon calculations in materials science. *Scripta Materialia* **2015**, *108*, 1–5.
